# *Nkx2.1*-derived astrocytes and neurons together with Slit2 are indispensable for anterior commissure formation

**DOI:** 10.1038/ncomms7887

**Published:** 2015-04-23

**Authors:** Shilpi Minocha, Delphine Valloton, Athena R. Ypsilanti, Hubert Fiumelli, Elizabeth A. Allen, Yuchio Yanagawa, Oscar Marin, Alain Chédotal, Jean-Pierre Hornung, Cécile Lebrand

**Affiliations:** 1 Department of Fundamental Neurosciences, University of Lausanne, Lausanne CH-1005, Switzerland; 2 INSERM UMR-S968, Institut de la Vision, Paris F-75012, France; 3 Sorbonne Université, UPMC University Paris 06, UMR_S 968, Institut de la Vision, Paris F-75012, France; 4 CNRS, UMR_7210, Paris F-75012, France; 5 Division of Biological and Environmental Sciences and Engineering, King Abdullah University of Science and Technology, Thuwal 23955, Saudi Arabia; 6 EPFL, Station 19, Batiment-SV, Room SV-2816, Lausanne CH-1015, Switzerland; 7 Department of Genetic and Behavioral Neuroscience, Gunma University Graduate School of Medicine and Japan Science and Technology Agency, CREST, Maebashi 371-8511, Japan; 8 Instituto de Neurociencias, Consejo Superior de Investigaciones Científicas and Universidad Miguel Hernández, Sant Joan d'Alacant 03550, Spain

## Abstract

Guidepost cells present at and surrounding the midline provide guidance cues that orient the growing axons through commissures. Here we show that the transcription factor Nkx2.1 known to control the specification of GABAergic interneurons also regulates the differentiation of astroglia and polydendrocytes within the mouse anterior commissure (AC). Nkx2.1-positive glia were found to originate from three germinal regions of the ventral telencephalon. Nkx2.1-derived glia were observed in and around the AC region by E14.5. Thereafter, a selective cell ablation strategy showed a synergistic role of Nkx2.1-derived cells, both GABAergic interneurons and astroglia, towards the proper formation of the AC. Finally, our results reveal that the Nkx2.1-regulated cells mediate AC axon guidance through the expression of the repellent cue, Slit2. These results bring forth interesting insights about the spatial and temporal origin of midline telencephalic glia, and highlight the importance of neurons and astroglia towards the formation of midline commissures.

## Abstract (web summary)

Guidepost cells provide guidance cues that orient growing axons in the brain but little is known about the midline guidepost cells that populate the mouse anterior commissure (AC). Here, the authors show that the transcription factor Nkx2.1 regulates the differentiation of astroglia and neurons that cooperate to guide AC axons through the expression of Slit2.

## Graphical abstract

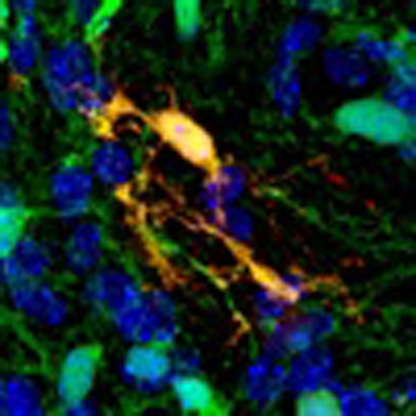

In the developing brain, axons succeed in navigating long distances and across a complex environment with the help of intermediate targets or guidepost cells that break the journey into smaller segments^1^. The current model for commissural axon guidance across the corpus callosum (CC) and the anterior commissure proposes that the glial cells localized around the CC, the AC and the fornix (F) express guidance factors that channel the axons into the correct path^1^,^2^,^3^,^4^,^5^,^6^,^7^,^8^,^9^,^10^,^11^. In addition, observations in mice and humans showed that two transient subpopulations of neurons, one glutamatergic and one GABAergic (γ-aminobutyric acidergic), occupy a strategic position at the CC midline and contribute to the guidance of growing callosal axons^12^,^13^,^14^,^15^. There is evidence that the radial glia that occupy the glial wedge at the border of the CC guide the commissural axons through Slit2-Robo1 signalling directing them towards and across the midline^11^,^16^. Also, the positioning of AC telencephalic projections is altered in Slit2 and Slit1/2 double knockout mice^5^,^6^,^8^. However, the cellular identity of the cells producing *Slits* around the AC is unknown.

During mouse neurogenesis, from E11 to E17, several transcription factors, including Tbr1 and Emx1, regulate the specification of pyramidal glutamatergic neurons in the dorsal telencephalon while others, such as Mash1, Dlx1/2, Nkx2.1 and Olig2, are responsible for the specification of GABAergic interneurons in the ventral telencephalon^17^,^18^,^19^,^20^,^21^,^22^. *Nkx2.1* is one of the earliest known genes to be expressed in the forebrain in a region that develops into the medial ganglionic eminence (MGE), its caudal part extending in the caudal ganglionic eminence, the anterior entopeduncular area (AEP), the anterior preoptic area (POA), the septum (SEP) and a part of the amygdala^19^,^23^,^24^,^25^. It has been shown to play a key role in embryos during the specification of GABAergic interneurons from the ventral telencephalon in addition to aiding in the production of the oligodendrocytes^17^,^20^,^21^,^24^,^26^,^27^.

It is generally accepted that astrocyte gliogenesis in the dorsal telencephalon occurs only after neurogenesis (after E17, in mice) when the radial glial cells of the dorsal pallium translocate their cell bodies towards the pial surface and differentiate into astrocytes in the cortex^28^,^29^,^30^,^31^,^32^,^33^. However, some reports have shown that some astrocytes at the CC midline are generated much earlier (between E13 and postnatal day 2 with a peak at E14; ref. 34). After birth, cortical astrocytes are believed to originate from migratory progenitors that reside in the dorsolateral subventricular zone (SVZ), and that, from this region, colonize the white matter and the cerebral cortex. The dorsolateral SVZ derives from Dlx2 precursors, and therefore may originate at first from the subpallium^35^. More recently, it has been shown that local differentiated glia in the mouse postnatal cortex constitute the major source of astrocytes instead of SVZ progenitors^36^. However, little is known about the precise time of genesis and origin of midline guidepost astroglia that populate the AC at early embryonic ages. Since several populations of glial cells are operating already during the embryonic period in guidance of forebrain commissures, a broader investigation of the potential sources of early glial cells is warranted.

Here, we show that early midline astroglia of the AC originate from the ventral telencephalon as early as E14.5. Therefore, we describe a step of astrogliogenesis that occurs in the ventral telencephalon concomitant with late neurogenesis, and generates astrocytes that occupy the AC. By using *in vitro* or *in utero* electroporation fate-mapping studies, and genetic lineage tracing, our analysis indicates that astrocytes are derived from Nkx2.1^+^ progenitors of the MGE, the AEP/POA and the septal nucleus. Since Nkx2.1 regulates GABAergic interneurons, astrocytes and polydendrocytes, we make use of selective cell ablation studies to discriminate between the potential guidepost functions of the three identified cell type populations. Interestingly, Nkx2.1-derived astrocytes are found, in addition to GABAergic neurons, to be necessary for the proper pathfinding of AC axons. Finally, our results utilizing *Nkx2.1-Cre*^*+*^*/Slit2*^*fl/fl*^ mice indicate that the *Nkx2.1*-regulated cells participate in the guidance of AC axons by expressing the repellent molecule Slit2. Thus, while confirming previous reports pointing towards a guidepost role for telencephalic glia, these results provide new insights for understanding the exact origin and function of these astrocytes in the axonal guidance of AC axons and the transcriptional regulation of these cells. This will give an additional insight on the genetic contribution and cellular mechanisms involved in abnormal commissural connections of the mammalian forebrain, including human patients.

## Results

### Nkx2.1-derived glia in the developing AC

**Figure 1:**
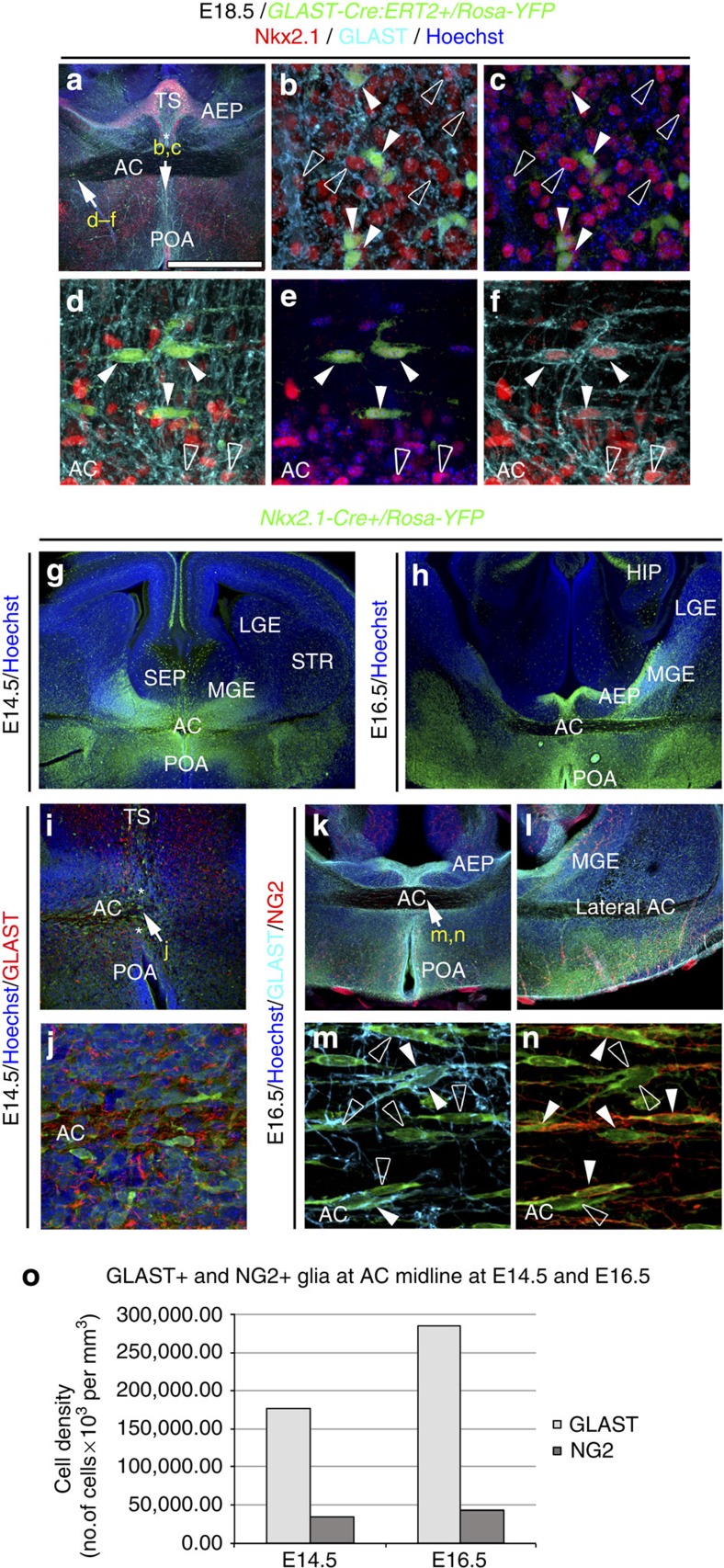
Presence of Nkx2.1-positive astrocytes in the AC during development. (**a**) Triple immunohistochemistry for YFP (green), Nkx2.1 (red) and GLAST (blue) in coronal AC sections from *GLAST-Cre:ERT2*^*+*^*/Rosa-YFP* mice at E18.5 (*n*=3). (**b**–**f**) Higher power views of the AC regions pointed by arrows in **a**. The Cre-mediated recombination was initiated under the control of the inducible GLAST promoter at E15, and the GLAST-derived cells were visualized (green) with the YFP signal. YFP^+^/GLAST^+^ astrocytes of the AC co-expressed Nkx2.1 (solid arrowheads **b**–**f**). Some of the GLAST^+^/Nkx2.1^+^ glia were not labelled by the YFP signal and might have been generated before the recombination was induced (open arrowheads in **b**–**f**). (**g**–**n**) Immunohistochemistry for YFP (**g**,**h**), for YFP and GLAST (**i**,**j**) and for YFP, NG2 and GLAST (**k**–**n**) in coronal sections from *Nkx2.1-Cre*^*+*^*/Rosa-YFP* mice at E14.5 (*n*=3; **g**,**i**,**j**) and at E16.5 (*n*=8; **h**,**k**–**n**). (**j**,**m**,**n**) High-magnified views of the regions indicated by an arrow in **i** and **k**. (**g**–**n**) From E14.5 to E16.5, the Cre-mediated recombination induced under the control of the Nkx2.1 promoter (visualized in green by the YFP) is visible in GLAST^+^ astrocytes and NG2^+^ polydendrocytes at the AC midline. (**k**–**n**) Cre-mediated recombination occurred in both GLAST^+^ (light blue) and NG2^+^ (red) glial cells that constitute two mutually exclusive populations of glia. Cell nuclei were counterstained in blue with Hoechst (**a**,**c**,**e**,**g**–**l**). HIP, hippocampus. The scale bar shown in panel **a** corresponds to the following length for panel(s) specified in brackets: 1,375 μm (**g**); 675 μm (**a**,**h**,**k**,**l**); 320 μm (**i**); 60 μm (**j**); 50 μm (**b**,**c**) and 40 μm (**d**–**f**,**m**,**n**). (**o**) Quantification of the GLAST^+^ and NG2^+^glial cells in *Nkx2.1-Cre*^*+*^*/Rosa-YFP* mice at E14.5 and E16.5. Bars (mean from *Nkx2.1-Cre*^*−*^*/Rosa-YFP* brains at E14.5 (*n*=3) and E16.5 (*n*=8)) represent the cell densities of GLAST^+^ and NG2^+^ glial cells at the AC midline. After quantification, the densities of GLAST+ and NG2+ glia were not significantly increased from E14.5 to E16.5 (*P*>0.05, Student's *t*-test).

**Figure 2:**
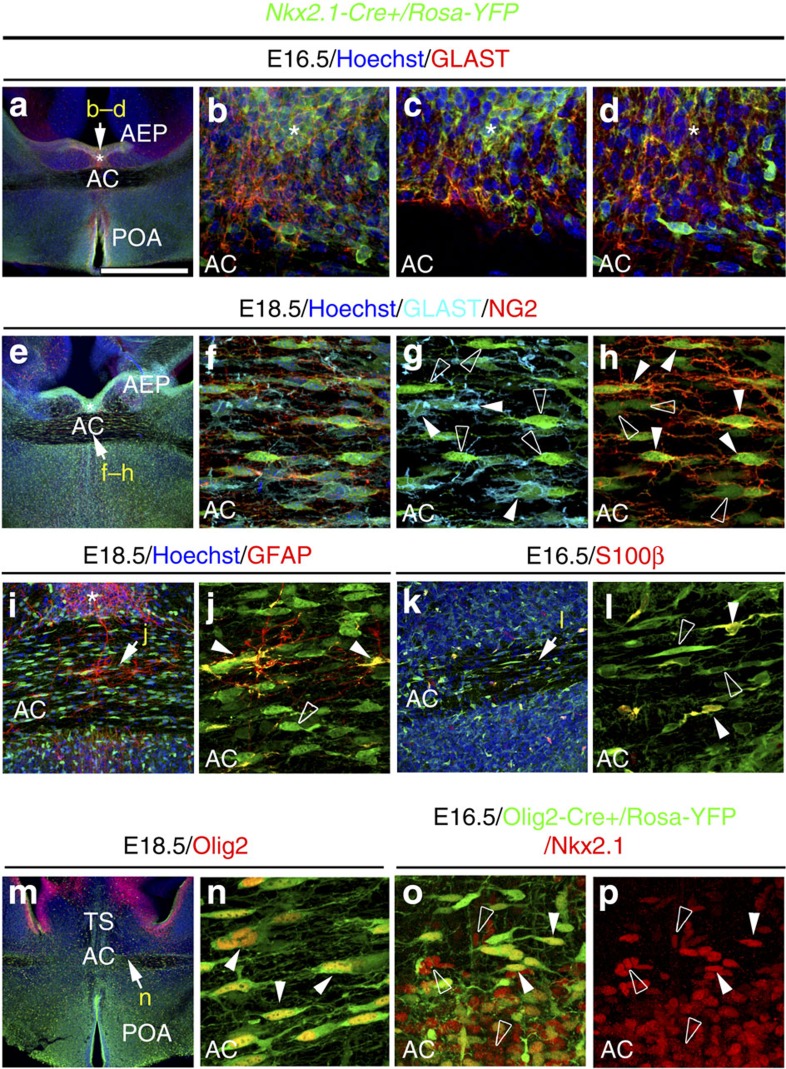
Different glial subpopulations are derived from Nkx2.1^+^ progenitors. (**a**–**d**,**i**–**n**) Double immunohistochemistry for YFP and GLAST (*n*=8; **a**–**d**), for YFP and GFAP (*n*=3; **i**–**j**), for YFP and S100β (*n*=3; **k**,**l**) and for YFP and Olig2 (*n*=3; **m**,**n**) on telencephalic coronal sections from *Nkx2.1-Cre*^*+*^*/Rosa-YFP* mice at E16.5 (**a**–**d**,**k**,**l**) and at E18.5 (**i**,**j**,**m**,**n**). Triple immunohistochemistry for YFP, GLAST and NG2 (*n*=3; **e**–**h**) in coronal sections from *Nkx2.1-Cre*^*+*^*/Rosa-YFP* mice at E18.5. (**b**–**d**,**f**–**h**,**j**,**l**,**n**) High-magnified views of the regions indicated by an arrow in **a**,**e**,**i**,**k** and **m**. (**c**,**d**) Individual planes of the Z-stack shown in **b**. (**a**–**n**) From E16.5 to E18.5, the Cre-mediated recombination induced under the control of the Nkx2.1 promoter (visualized in green by the YFP) is visible in GLAST^+^ and GFAP^+^ astroglia of the AC white matter and of the tunnel region (*) (**a**–**d**,**e**–**g**,**i**,**j**), in all NG2^+^ polydendrocytes of the AC (**e**,**f**,**h**), in all S100β^+^ (**k**,**l**) and many Olig2^+^ glial cells of the AC (**m**,**n**). (**e**–**h**) GLAST^+^ (light blue; solid arrowheads in **g**) and NG2^+^ (red; solid arrowheads in **h**) glial cells constitute two mutually exclusive populations of glia. Colocalization between the green and the red channel is highlighted in yellow. (**o**,**p**) Immunohistochemistry for YFP and Nkx2.1 in coronal sections from *Olig2-Cre*^*+*^*/Rosa-YFP* mice at E16.5 (*n*=3). YFP^+^ cells of the AC co-expressed Nkx2.1 (solid arrowheads in **o**,**p**). Some of the Nkx2.1^+^ cells were not labelled by the YFP signal (open arrowheads in **o**,**p**). Cell nuclei were counterstained in blue with Hoechst (**a**–**f**,**i**,**k**,**m**). The scale bar shown in panel **a** corresponds to the following length for panel(s) specified in brackets: 675 μm (**a**,**e**,**m**); 160 μm (**i**,**k**); 60 μm (**l**,**o**,**p**); 55 μm (**b**–**d**); 50 μm (**f**–**h**,**j**,**n**).

**Figure 3:**
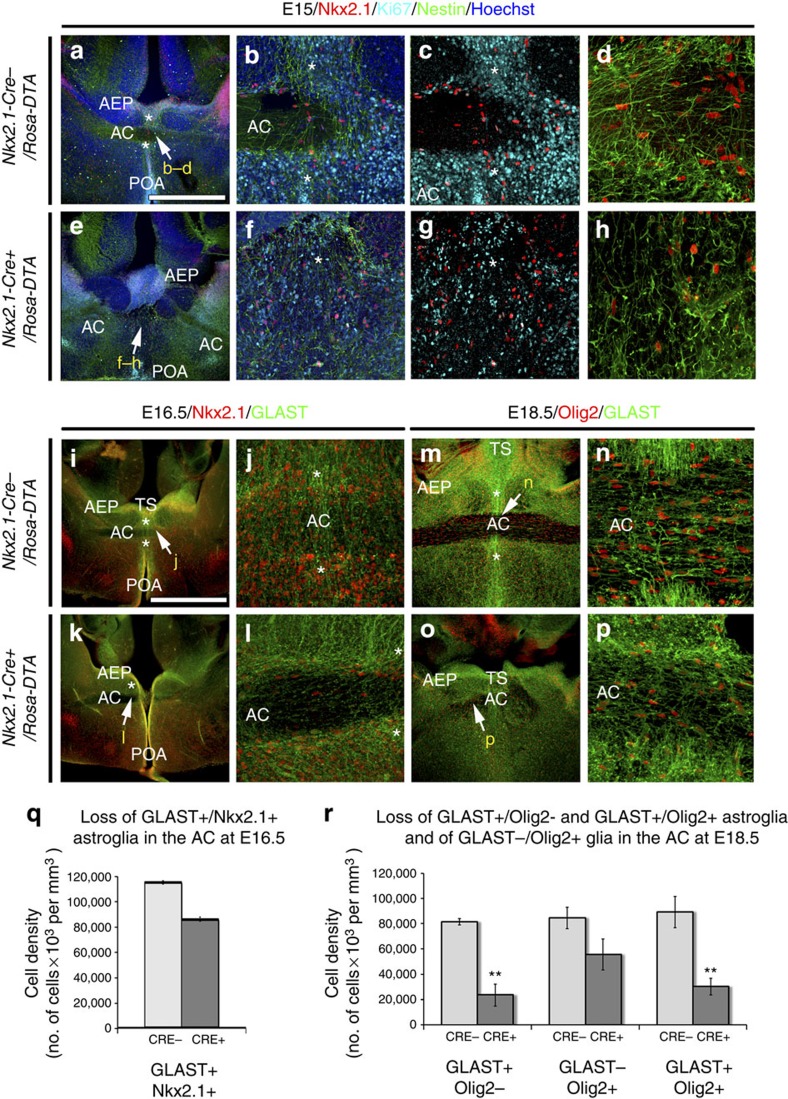
Post-mitotic glia of the AC are ablated in *Nkx2.1-Cre*^*+*^*/Rosa-DTA* brains. (**a**–**h**) Triple immunochemistry for Nkx2.1, Ki67 and Nestin on E15 coronal slices from *Nkx2.1-Cre*^*−*^*/Rosa-DTA* (*n*=3; **a**–**d**) and *Nkx2.1-Cre*^*+*^*/Rosa-DTA* (*n*=3; **e**–**h**) mice. (**i**–**p**) Double immunochemistry for Nkx2.1 and GLAST on E16.5 (**i**–**l**) and for Olig2 and GLAST on E18.5 (**m**–**p**) coronal slices from *Nkx2.1-Cre*^*−*^*/Rosa-DTA* (**i**,**j**,**m**,**n**) and *Nkx2.1-Cre*^*+*^*/Rosa-DTA* (**k**,**l**,**o**,**p**) mice. (**b**–**d**,**f**–**h**,**j**,**l**,**n**,**p**) High-magnified views of the regions indicated by an arrow in **a**,**e**,**i**,**k**,**m** and **o**. (**a**–**l**) The GLAST^+^/Nestin^+^/Nkx2.1^+^/Ki67^−^ post-mitotic tunnel astroglia forming a palisade (*) surrounding the AC are depleted in the *Nkx2.1-Cre*^*+*^*/Rosa-DTA* (**e**–**h**,**k**,**l**) compared with control (**a**–**d**,**i**,**j**) mice. (**m**,**n**) In control mice, numerous GLAST^+^/Olig2^−^, GLAST^−^/Olig2^+^ and GLAST^+^/Olig2^+^ glial cells are observed within and around the AC. (**o**,**p**) In *Nkx2.1-Cre*^*+*^*/Rosa-DTA* mice, the number of AC GLAST^+^/Olig2^−^, GLAST^−^/Olig2^+^ and GLAST^+^/Olig2^+^ is decreased after cell ablation. Cell nuclei were counterstained in blue with Hoechst (**a**,**b**,**e**,**f**). The scale bar shown in panel **a** corresponds to the following length for panel(s) specified in brackets: 675 μm in **a**,**e**,**i**,**k**,**m**,**o**; 160 μm (**b**,**c**,**f**,**g**,**j**,**l**,**n**,**p**); 50 μm (**d**,**h**). (**q**) Bars (means±s.e.m.) from *Nkx2.1-Cre*^*−*^*/Rosa-DTA* (*n*=5) and *Nkx2.1-Cre*^*+*^*/Rosa-DTA* (*n*=3) brains represent the cell densities of GLAST^+^/Nkx2.1^+^ at the AC midline at E16.5. After quantification, the density of GLAST^+^ astrocytes expressing Nkx2.1 was decreased in *Nkx2.1-Cre*^*+*^*/Rosa-DTA* mice compared with control mice. (**r**) Bars (means±s.e.m.) from *Nkx2.1-Cre*^*−*^*/Rosa-DTA* (*n*=5) and *Nkx2.1-Cre*^*+*^*/Rosa-DTA* (*n*=5) brains represent the cell densities of GLAST^+^/Olig2^−^, GLAST^−^/Olig2^+^ and GLAST^+^/Olig2^+^ glial cells at the AC midline at E18.5. After quantification, the density of GLAST^+^ astrocytes expressing or not Olig2 was significantly decreased in *Nkx2.1-Cre*^*+*^*/Rosa-DTA* mice compared with control mice (**P<0.01, *n*=5, Student's *t*-test).

We found numerous Nkx2.1^+^ astrocytes in the AC white matter and in the tunnel region surrounding it from E14.5 to E18.5. They expressed markers such as Nestin, glutamate aspartate transporter (GLAST) and glial fibrillary acidic protein (GFAP) that are specific for post-mitotic astrocytes in the AC (Fig. 1 and Supplementary Fig. 1a–c). Using tamoxifen-inducible *GLAST-Cre:ERT2+/Rosa26-YFP* mice, early astrocytes were traced outside germinal zones and within the midline AC (Fig. 1). Many of the early astrocytes visualized by the yellow fluorescent protein (YFP) and GLAST staining co-expressed Nkx2.1 (Fig. 1, solid arrowheads in b–f). These results indicated that probably Nkx2.1 regulates the specification of the GLAST-derived YFP^+^ astrocytes, and that some astrocytes were generated at E14.5 in the ventral telencephalon when the recombination was induced. Next, by using *Nkx2.1-Cre+/Rosa-YFP* reporter mice, we verified- the existence of *Nkx2.1*-derived YFP^+^ astrocytes expressing GLAST or GFAP within the AC white matter and the tunnel region (*) beginning E14.5 (Figs 1i–m,o and 2a–j, solid arrowheads in Fig. 2g,j). Interestingly, we observed that the YFP signal was also detected in a GLAST^+^ population of astrocytes in the AC, which co-expressed Olig2, and were different from the GLAST^+^ astrocytes previously described (Fig. 3r).

We also observed the presence of *Nkx2.1*-derived YFP^+^ polydendrocytes expressing nerve/glial antigen 2 (NG2) throughout the AC white matter starting from E14.5 (Figs 1k,l,n and 2h, solid arrowheads in Fig. 1n and Fig. 2h). NG2 is a chondroitin sulfate proteoglycan expressed by polydendrocytes that are considered as oligodendrocyte precursor cells and constitute a population of cells different from neurons, mature oligodendrocytes, astrocytes and microglia^37^,^38^. In the AC of *Nkx2.1-Cre*^*+*^*/Rosa-YFP* embryos, the YFP signal was expressed in both GLAST^+^ astrocytes and NG2^+^ polydendrocytes; importantly, both populations were exclusively non-overlapping (Figs 1m,n and 2g,h). Complementary analyses using *NG2-Cre*^*+*^*/Rosa-YFP* mice showed that all YFP^+^ polydendrocytes were Olig2^+^, the majority were also S100β^+^ and none were Nkx2.1^+^ (Supplementary Fig. 1a–i). This indicates that part of the *Nkx2.1*-derived Olig2^+^ and S100β^+^ cells with downregulated Nkx2.1 correspond to polydendrocytes (Fig. 2k–p). Therefore, all *Nkx2.1*-derived NG2^+^ polydendrocytes expressed Olig2, while only some *Nkx2.1*-derived GLAST^+^ astrocytes expressed Olig2. Moreover, many *Nkx2.1*-derived NG2^+^ polydendrocytes, but only few GLAST^+^ astrocytes, expressed S100β. Hence, the *Nkx2.1*-derived glial cells can be divided into the following four major categories: astrocyte-like glial cells that are either GLAST^+^/GFAP^+^/Olig2^−^ or GLAST^+^/GFAP^−^/Olig2^+^, or polydendrocyte-like cells that are either NG2^+^/Olig2^+^/S100β^+^ or NG2^+^/Olig2^+^/S100β^−^ (Supplementary Fig. 2). Only the Nkx2.1-derived astrocytes continued to express the Nkx2.1 protein after they had left the ventricular zone.

To identify the timing of occurrence of the Nkx2.1-derived cells in the AC and the surrounding tunnel region, we analysed *Nkx2.1-Cre*^*+*^*/Rosa-YFP* embryos beginning at E14.5. Interestingly, already by E14.5, the Nkx2.1-derived astrocytes and polydendrocytes were observed to be present at the AC midline centre (Fig. 1g,i,j,o). At E15, labelling with cell cycle marker, Ki67 unravelled the presence of numerous non-dividing Nkx2.1^+^ astrocytes co-stained with Nestin in the AC and the surrounding tunnel zone (*) (Fig. 3a–d). Hence, the generation and subsequent occurrence of Nkx2.1-derived astrocytes and polydendrocytes precedes AC axonal crossing. Later analysis at E16.5 and E18.5 revealed continued presence of Nkx2.1-derived glia in and around the AC (Figs 1h,k–n and 2a–n). Many Nkx2.1^+^ progenitor cells in the AEP and POA germinal sites adjacent to the tunnel region (*) were found to be positive for Ki67 expression from E14.5 to E16.5 (Fig. 3a–c). Hence, these results point towards continued production of Nkx2.1-derived cells from the surrounding precursor regions. Furthermore, following 5-bromo-2′-deoxy-uridine (BrdU) injections, we observed that the Nkx2.1^+^/GLAST^+^/GFAP^+^ embryonic astrocytes of the AC were primarily generated between E14.5 and E16.5. Considerable staining was visualized when BrdU was injected at E14.5 and E16.5, and brains were harvested at E18.5 for analysing BrdU incorporation (Supplementary Fig. 3).

Next, to ascertain the survival of the embryonic *Nkx2.1*-derived glial cells, *Nkx2.1-Cre*^*+*^*/Rosa-YFP* mice were analysed at various early and late postnatal ages. Immunostaining was performed with antibodies against different glial markers, namely, GLAST, GFAP, S100β, Olig2 and NG2 (Supplementary Fig. 4). Until P14, both types of Nkx2.1-derived glia positively co-stained with either of the tested glial markers were still found in the AC (Supplementary Fig. 4a–j). However, by P30, a significant reduction in the number of NG2^+^ Nkx2.1-derived polydendrocytes could be seen (Supplementary Fig. 4o). Subsequent analysis in adult mice at P73 revealed a total absence of Nkx2.1-derived cells co-stained with NG2 and a drastic decrease in those positive for Olig2 (Supplementary Fig. 4s–t). The GFAP^+^ and S100β^+^ Nkx2.1-derived astrocytes continued to prevail (Supplementary Fig. 4p–r). Hence, the Nkx2.1-derived oligodendroglia and polydendrocytes constitute a transient population, whereas the Nkx2.1-derived astroglia persist until adulthood.

Our results, therefore, demonstrate that in embryos, different embryonic astrocyte-like or polydendrocyte-like glial cell subpopulations occupy the AC region and are derived from Nkx2.1^+^ progenitors of the ventral telencephalon. Nkx2.1-derived polydendrocytes are transient, whereas astrocytes survive until adulthood, hence suggesting different roles for the two populations during development. Also, since Nkx2.1-derived cells occupy the AC midline region before axonal fibre crossing, they might play a role in the guidance of AC axons.

### Ventral telencephalic origin of Nkx2.1-derived astrocytes

**Figure 4:**
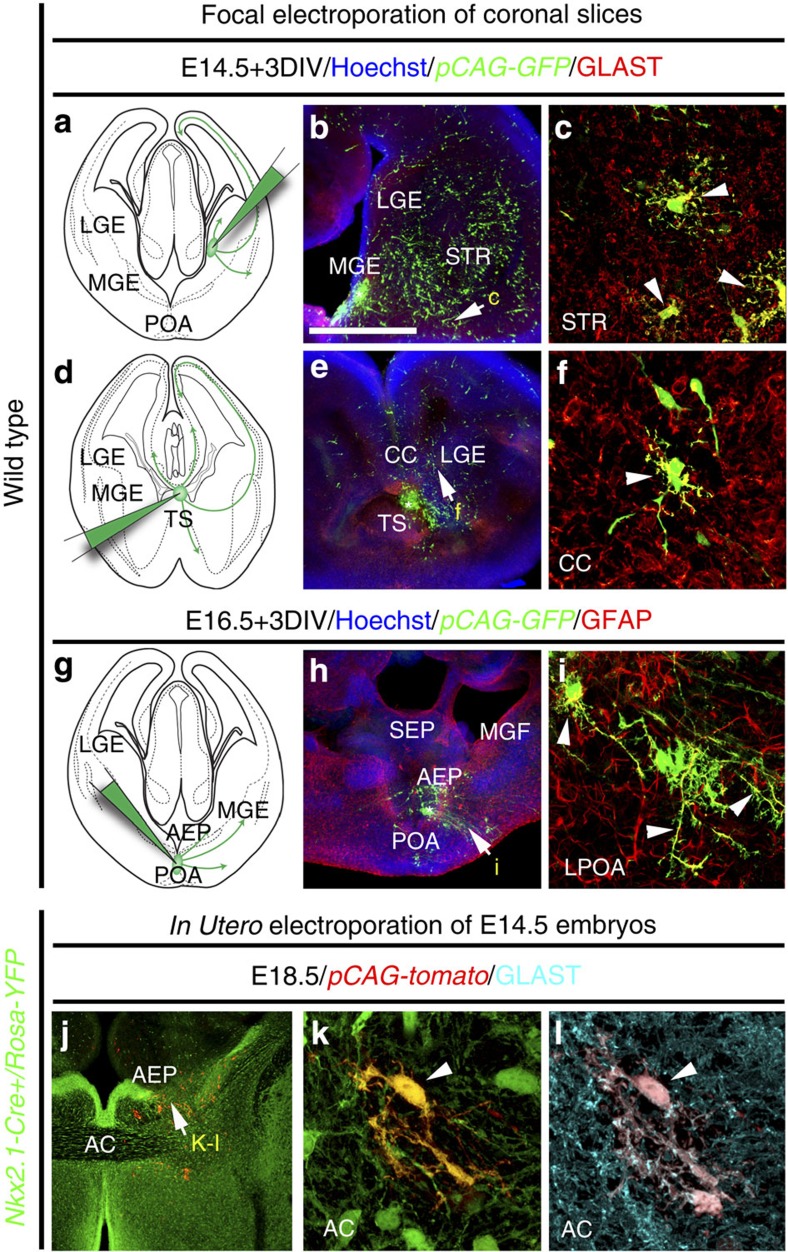
*Nkx2.1*-derived subpallial domains give rise to embryonic astroglia. (**a**–**i**) Use of *in vitro* electroporation to determine whether embryonic astrocytes originate from subpallial sites. The MGE (*n*=12; **a**–**c**), TS (*n*=7; **d**–**f**) and the POA (*n*=6; **g**–**i**) of WT E14.5–E16.5 coronal telencephalic slices were focally electroporated with a plasmid (*pCAG-EGFP)* expressing the EGFP. Double immunochemistry for GFP and GLAST (**b**,**c**,**e**,**f**), and for GFP and GFAP (**h**,**i**) was performed. (**c**,**f**,**i**) High-magnified views of the regions indicated by an arrow in **b**,**e**,**h**, respectively. (**a**–**f**) After focal electroporation, subpallial progenitors gave rise to GFP^+^/GLAST^+^ astrocytes that have migrated in the striatal mantle (STR) and the CC (solid arrowheads in **c**,**f**). (**g**–**i**) A similar electroporation into the POA, gave rise to GFP^+^/GFAP^+^ astroglia that have migrated to the lateral part of the POA (LPOA; solid arrowheads in **i**). (**j**–**l**) Experimental paradigms used to ascertain the Nkx2.1-derived origin of embryonic astroglia. To this end, a Tomato expressing plasmid (*pCAG-tomato*) was electroporated into the subpallial domain of *Nkx2.1-Cre*^*+*^*/Rosa-YFP* embryos at E14.5 and triple immunohistochemistry for YFP, tomato and GLAST was performed (*n*=8). (**j**–**l**) After *in utero* electroporation, progenitors of the AEP gave rise to several YFP^+^ Nkx2.1-derived astroglia, labelled for tomato and GLAST, that migrate to the AC. Cell nuclei were counterstained in blue with Hoechst (**b**,**e**,**h**). Colocalization between the green and the red channel is highlighted in yellow (**c**,**f**,**i**,**k**). The scale bar shown in panel **a** corresponds to the following length for panel(s) specified in brackets: 675 μm (**b**,**e**,**h**,**j**); 60 μm (**c**,**f**,**i**); 40 μm (**k**,**l**).

As early as E14.5, the radial glial precursors of the MGE and the AEP/POA ventricular zone co-expressed Nkx2.1 and Nestin or GLAST, and by E16.5, they co-expressed Nkx2.1 and GFAP (Fig. 3a–c and Supplementary Fig. 5a–f). Surprisingly, a third region localized in the SEP, the triangular septal nucleus (TS), also contained numerous Nkx2.1^+^ progenitors expressing astroglial markers (Supplementary Fig. 5a,d). Moreover, the basal progenitors in the SVZ of the MGE co-expressed the same markers (Supplementary Fig. 5a–f). It is generally accepted that cells expressing astroglial markers are differentiated astrocytes as soon as they escape the germinal ventricular and SVZs^29^,^32^,^39^. Interestingly, numerous mature Nkx2.1^+^ astrocytes expressing GLAST or GFAP were found in the ganglionic mantle zone, as well as in the lateral part of the POA far from the germinal zones (Supplementary Fig. 5, arrowheads in c and f). In addition, numerous Nkx2.1^+^/Nestin^+^/Ki67^−^ astrocytes originating from the MGE or the AEP/POA were dispersed in the AC region (Fig. 3a–c). Analysis of the *GLAST-Cre:ERT2*^*+*^*/Rosa-YFP* mice confirmed that early astrocytes co-expressing Nkx2.1 and GLAST originate from the MGE, the AEP/POA and the TS (Supplementary Fig. 1j–o). These cells also migrated to the parenchymal regions such as the AC and the striatum (Fig. 1 and Supplementary Fig. 5g–i). In addition, the origin of Nkx2.1^+^ astroglia was examined by combining immunostaining for glial markers with focal *ex vivo* or *in utero* electroporation (Fig. 4). Although slice and *in utero* electroporation are effective methods for administering plasmids into brain sections or entire brain, respectively, both of them are subject to technical limitations hindering possibility of targeting the same subregion in all experiments. Hence, we only chose slices or brains where we were convinced that the site of electroporation was focally restricted after careful investigation of all sections. In E14.5 and E16.5 embryonic wild-type (WT) brain slices, electroporation of the *pCAG-GFP* plasmid into the MGE, the TS or the AEP/POA gave rise to GLAST^+^ or GFAP^+^ astrocytes of ventral telencephalic regions (Fig. 4a–i). Furthermore, we also confirmed our results by *in utero* electroporation of the *pCAG-tomato* plasmid into the ventral telencephalon of E14.5 *Nkx2.1-Cre*^*+*^*/Rosa-YFP* embryos; indeed, many *Nkx2.1*-derived YFP^+^ and GLAST^+^ astrocytes labelled for the tomato could be seen migrating from the AEP region to the AC and striatum mantle zone (Fig. 4j–l).

These results together with the fate-mapping analysis indicate that the embryonic *Nkx2.1*-derived astroglia of the AC originate and migrate from the following three potential distinct Nkx2.1^*+*^ domains of the ventral telencephalon: the MGE, the AEP/POA and the TS.

### Nkx2.1-derived cells cooperate to guide AC axons

**Figure 5:**
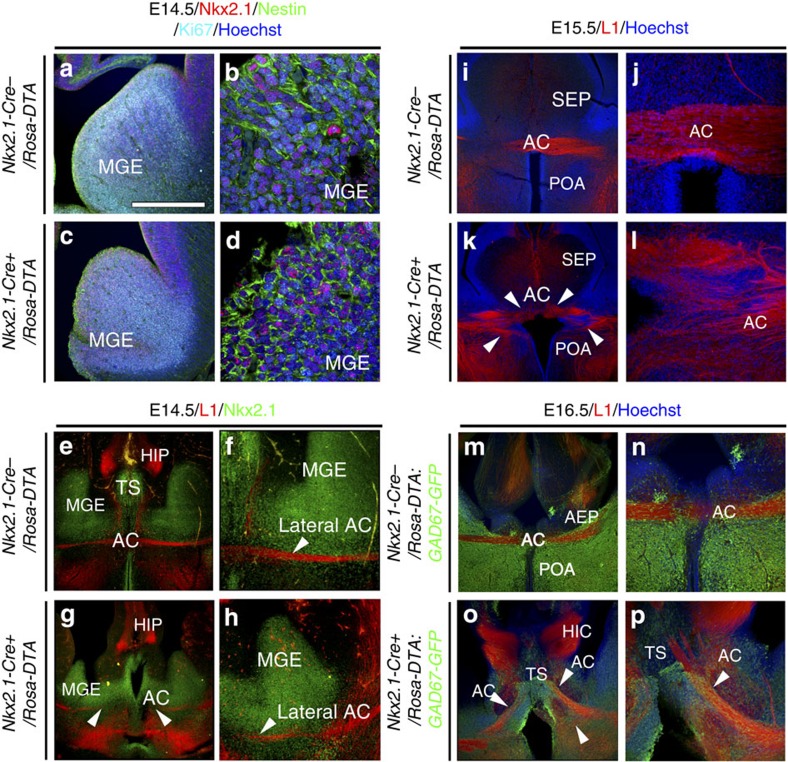
Early midline guidance defects of the AC in *Nkx2.1-Cre*^*+*^*/Rosa-DTA* mice. (**a**–**h**) Triple immunochemistry for Nkx2.1, Ki67 and Nestin (*n*=4; **a**–**d**) and double immunochemistry for L1 and Nkx2.1 (*n*=3; **e**–**h**) on E14.5 coronal slices from *Nkx2.1-Cre*^*−*^*/Rosa-DTA* (**a**,**b**,**e**,**f**) and *Nkx2.1-Cre*^*+*^*/Rosa-DTA* (**c**,**d**,**g**,**h**) mice. (**b**,**d**) High-magnified views of MGE seen in **a**,**c**, respectively. (**f**,**h**) Higher power view of MGE and the lateral side of the AC seen in **e**,**g**, respectively. (**a**–**d**) Labelling with the cell cycle marker Ki67, combined with Nestin, revealed active proliferation of Nkx2.1^+^ progenitors in the MGE precursor regions in both control (**a**,**b**) and mutant (**c**,**d**) brains. (**e**–**h**) At E14.5, we could clearly see that in control (**e**,**f**) and mutant (**g**,**h**) brains, the L1-positive tracts behaved similarly within the lateral part of the AC. (**i**–**l**) Immunochemistry for L1 in coronal sections from control *Nkx2.1-Cre*^*−*^*/Rosa-DTA* (*n*=3; **i**,**j**) and *Nkx2.1-Cre*^*+*^*/Rosa-DTA* (*n*=3; **k**,**l**) mice at E15.5. (**j**,**l)** High-magnified views of the AC midline seen in **i**,**k**, respectively. (**i**,**j**) In *Nkx2.1-Cre*^*−*^*/Rosa-DTA* control mice, L1^+^ commissural axons crossed the AC midline and grew towards the contralateral cortex. (**k**,**l**) By contrast, in mutant *Nkx2.1-Cre*^*+*^*/Rosa-DTA* mice, defasciculation of axonal tracts at the AC midline can be seen and axons do not cross the midline (solid arrowheads). (**m**–**p**) Double immunochemistry for green fluorescent protein (GFP) and L1 on coronal sections from control *Nkx2.1-Cre*^*−*^*/Rosa-DTA:GAD67-GFP* (*n*=3; **m**,**n**) and *Nkx2.1-Cre*^*+*^*/Rosa-DTA:GAD67-GFP* (*n*=3; **o**,**p**) mice at E16.5. (**n**,**p**) High-magnified views of the AC midline seen in **m**,**o**, respectively. (**o**,**p**) In *Nkx2.1-Cre*^*+*^*/Rosa-DTA:GAD67-GFP* mice, there was a severe disorganization of GAD67-GFP^+^ interneurons. At E16.5, axons of the AC still did not cross the midline and are separated into two tracts that point ventrally and dorsally in the TS. HIC, hippocampal commissure; HIP, hippocampus. The scale bar shown in panel **a** corresponds to the following length for panel(s) specified in brackets: 675 μm (**a**,**c**,**e**,**g**,**i**,**k**,**m**,**o**); 320 μm (**f**,**h**,**n**,**p**); 160 μm (**j**,**l**); 50 μm (**b**,**d**).

In order to assess specifically the potential involvement of the *Nkx2.1*-derived glia or neurons in axon guidance, we made use of a cell ablation strategy. The *Nkx2.1-Cre* mice were crossed with *Rosa-DTA* mice^40^ to generate embryos that expressed the highly potent diphtheria toxin fragment A (DTA) under the control of the Nkx2.1 promoter. The DTA expression leads to the selective ablation of *Nkx2.1*-derived post-mitotic cells in the *Nkx2.1-Cre*^*+*^/*Rosa-DTA* mutant embryos. In this process, the Nkx2.1^+^ precursors were left unaffected since the Nkx2.1-dependent expression and action of the diphtheria toxin took several days. Embryos beginning from E14.5 were analysed to ascertain the development of precursor regions and the effect of the toxin on Nkx2.1-derived cell population. At E14.5, comparable labelling for Ki67 and Nestin was observed in the MGE precursors region of control *Nkx2.1-Cre*^*−*^/*Rosa-DTA* and conditional mutant *Nkx2.1-Cre*^*+*^/*Rosa-DTA* brains (Fig. 5a–d), thus confirming their active proliferation. Although the MGE was formed in both, it appeared to be slightly smaller in *Nkx2.1-Cre*^*+*^/*Rosa-DTA* embryos when compared with littermate controls (compare Fig. 5a with Fig. 5c). The small-sized MGE in mutant *Nkx2.1-Cre*^*+*^/*Rosa-DTA* embryos could be due to increased incidence of cell death, as visualized by staining for cleaved caspase 3 (Casp3; Supplementary Fig. 6). Labelling with Casp3 revealed cell death of Nkx2.1^+^ progenitors in the MGE precursor regions in both control and mutants at E14.5 (Supplementary Fig. 6a–c). Also, we could observe increased Casp3 labelling in the AC region in the mutants (Supplementary Fig. 6d). The increased cell death was spread out between E14.5 and E16.5 since the diphtheria toxin took time to accumulate (Supplementary Fig. 6e–h). On the contrary, in the *Nkx2.1*^*−/−*^ mice, the early development of ventral telencephalon area is impaired and precludes the study of the AC formation. In *Nkx2.1*^*−/−*^ mice, the MGE is re-specified to a more dorsal structure, the lateral ganglionic eminence (LGE)^20^. Thus, we utilized only *Nkx2.1-Cre*^*+*^/*Rosa-DTA* mice to understand the phenotype resulting specifically from the loss of Nkx2.1-derived cells at the AC without affecting the progenitor zones.

**Figure 6:**
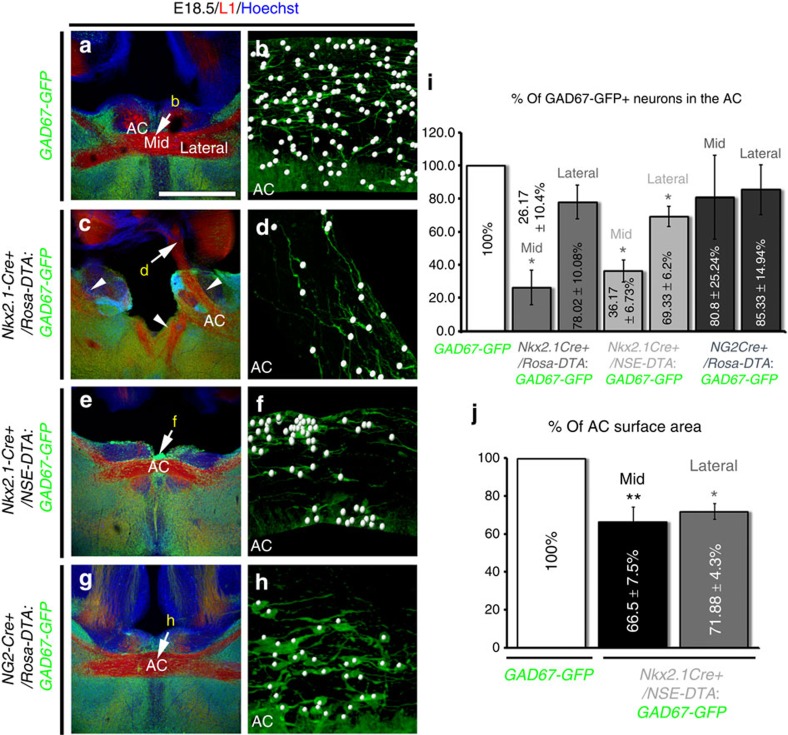
Loss of *GAD67-GFP*^*+*^ interneurons and axonal guidance defects. (**a**–**h**) Double immunohistochemistry for GFP and L1 on AC coronal sections from *GAD67-GFP* (*n*=3; **a**,**b**), *Nkx2.1-Cre*^*+*^*/Rosa-DTA:GAD67-GFP* (*n*=4; **c**,**d**), *Nkx2.1-Cre*^*+*^*/NSE-DTA:GAD67-GFP* (*n*=4; **e**,**f**) and *NG2-Cre*^*+*^*/Rosa-DTA:GAD67-GFP* (*n*=3; **g**,**h**) mice brains at E18.5. Cell nuclei were counterstained in blue with Hoechst (**a**,**c**,**e**,**g**). (**b**,**d**,**f**,**h**) Higher power views showing the quantification of the GAD67-GFP^+^ interneurons (white spots) within the AC pointed by an arrow in **a**,**c**,**e**,**g**, respectively. (**a**,**b**) In the AC of *GAD67-GFP* mice, numerous GAD67-GFP^+^ interneurons (green) populated the midline. (**c**,**d**) In *Nkx2.1-Cre*^*+*^*/Rosa-DTA:GAD67-GFP* mice, the GAD67-GFP^+^ interneurons were drastically depleted from the AC (compare **d** with **b**). (**e**,**f**) In *Nkx2.1-Cre*^*+*^*/NSE-DTA:GAD67-GFP* mice, the GAD67-GFP^+^ interneurons were also strongly depleted from the AC (compare **f** with **b**). (**g**,**h**) In *NG2-Cre*^*+*^*/Rosa-DTA:GAD67-GFP* mice, the GAD67-GFP^+^ interneurons were similar in number to those observed in the WT mice in the AC (compare **h** with **b**). (**i**) Bars (mean±s.e.m. from *GAD67-GFP* (*n*=3)*, Nkx2.1-Cre*^*+*^*/Rosa-DTA:GAD67-GFP* (*n*=4)*, Nkx2.1-Cre*^*+*^*/NSE-DTA:GAD67-GFP* (*n*=4) and *NG2-Cre*^*+*^*/Rosa-DTA:GAD67-GFP* (*n*=3)) represent the percentage of GAD67-GFP^+^ interneurons, in the middle (mid) and on the lateral sides (lateral) of the AC in control *GAD67-GFP* mice compared with *Nkx2.1-Cre*^*+*^*/Rosa-DTA:GAD67-GFP, Nkx2.1-Cre*^*+*^*/NSE-DTA:GAD67-GFP* and *NG2-Cre*^*+*^*/Rosa-DTA:GAD67-GFP* mice. The results show that there is a drastic and significant decrease of GAD67-GFP^+^ neurons (which is more pronounced in the middle than on the lateral sides) in *Nkx2.1-Cre*^*+*^*/Rosa-DTA:GAD67-GFP* and *Nkx2.1-Cre*^*+*^*/NSE-DTA:GAD67-GFP* mice compared with the WT mice (*P<0.05, Student's *t*-test). (**j**) Bars (mean±s.e.m. from *GAD67-GFP* (*n*=7) and *Nkx2.1-Cre*^*+*^*/NSE-DTA:GAD67-GFP* (*n*=5)) represent the AC surface area in the *Nkx2.1-Cre*^*+*^*/NSE-DTA:GAD67-GFP* mutant compared with control *GAD67-GFP* mice. Quantification reveals a significant reduction of the AC surface area in the mutants compared with WT mice (**P<0.01, *P<0.05, Student's *t*-test). The scale bar shown in panel **a** corresponds to the following length for panel(s) specified in brackets: 675 μm (**a**,**c**,**e**,**g**); 160 μm (**b**,**d**,**f**,**h**).

**Figure 7:**
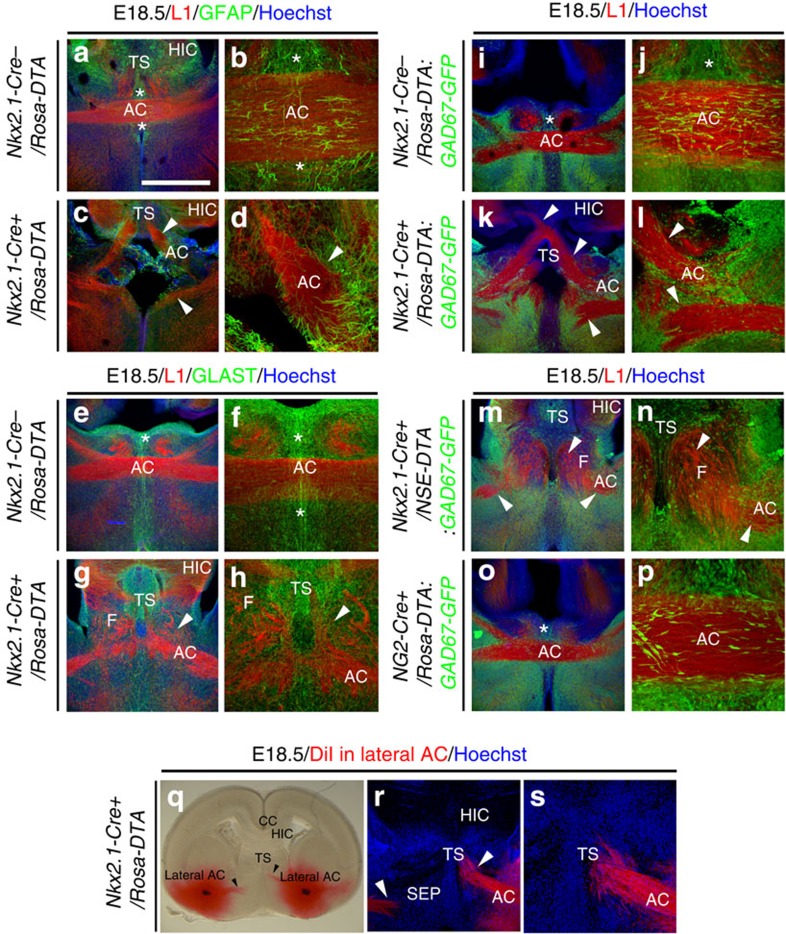
Midline guidance defects of the AC axons in E18.5 *Nkx2.1-Cre*^*+*^*/Rosa-DTA* mice. (**a**–**h**) Double immunochemistry for L1 and GFAP (*n*=3; **a**–**d**) and for L1 and GLAST (*n*=3; **e**–**h**) in coronal sections from control *Nkx2.1-Cre*^*−*^*/Rosa-DTA* (**a**,**b**,**e**,**f**) and *Nkx2.1-Cre*^*+*^*/Rosa-DTA* (**c**,**d**,**g**,**h**) mice at E18.5. (**b**,**d**,**f**,**h**) High-magnified views of the AC midline seen in **a**,**c**,**e**,**g**, respectively. (**a**,**b**,**e**,**f**) In *Nkx2.1-Cre*^*−*^*/Rosa-DTA* control mice, L1^+^ commissural axons crossed the AC midline and grew towards the contralateral cortex. (**c**,**d**,**g**,**h**) By contrast, in the mutant *Nkx2.1-Cre*^*+*^*/Rosa-DTA* mice, the majority of AC axons did not cross the midline and form two large ectopic bundles of axons on either ipsilateral side of it (solid arrowheads). The axons of the dorsal tract were misrouted into the TS or into the fornix (**F**). Some midline GFAP^+^ and GLAST^+^ astrocytes were missing and those that remained were mispositioned in the mutants. (**i**–**p**) Double immunochemistry for GFP and L1 on coronal sections from control *Nkx2.1-Cre*^*−*^*/Rosa-DTA:GAD67-GFP* (*n*=3; **i**,**j**), *Nkx2.1-Cre*^*+*^*/Rosa-DTA:GAD67-GFP* (*n*=4; **k**,**l**), *Nkx2.1-Cre*^*+*^*/NSE-DTA:GAD67-GFP* (*n*=4; **m**,**n**) and *NG2-Cre*^*+*^*/Rosa-DTA:GAD67-GFP* (*n*=3; **o**,**p**) mice at E18.5. (**j**,**l**,**n**,**p**) High-magnified views of the AC midline seen in **i**,**k**,**m**,**o**, respectively. (**i**,**j**) In control *Nkx2.1-Cre*^*−*^*/Rosa-DTA:GAD67-GFP* mice, GAD67-GFP^+^ interneurons were observed within the white matter and around the AC. (**k**,**l**) In *Nkx2.1-Cre*^*+*^*/Rosa-DTA:GAD67-GFP* mice, there was a severe loss of GAD67-GFP^+^ interneurons. (**m**,**n**) In *Nkx2.1-Cre*^*+*^*/NSE-DTA:GAD67-GFP* mice, GAD67-GFP^+^ interneurons were depleted in the AC. Part of the axons projected anteriorly and intermixed with axons of the fornix forming aberrant bundles. (**o**,**p**) In *NG2-Cre*^*+*^*/Rosa-DTA:GAD67-GFP* mice, no axonal guidance defects of commissural axons were observed. (**q**–**s**) Crystals of a lipophilic dye DiI were placed in the lateral sides of the AC (lateral AC) of coronal sections from *Nkx2.1-Cre*^*+*^*/Rosa-DTA* mice (*n*=11) at E18.5. (**r**,**s**) High-magnified views of the AC midline seen in **q**. In the mutant brains, the trajectory of several AC axonal tracts was perturbed, with ipsilateral projections oriented rostro-dorsally towards the TS or ventrally in the SEP. HIC, hippocampal commissure. The scale bar shown in panel **a** corresponds to the following length for panel(s) specified in brackets: 2,700 μm (**q**); 675 μm (**a**,**c**,**e**,**g**,**i**,**k**,**m**,**o**,**r**); 320 μm (**f**,**h**,**s**); 160 μm (**b**,**d**,**j**,**l**,**n**,**p**).

Since, the Nkx2.1-derived cell population comprised neurons and glia, we assayed the effect of cell ablation strategy on both of these populations. The *Nkx2.1-Cre*^*+*^*/Rosa-DTA:GAD67-GFP* mice displayed a loss of >70% GAD67-GFP^+^ interneurons at the AC midline (Fig. 6c,d,i). The Nkx2.1^+^ ‘tunnel-like' astroglia of the AC (*) were seen to originate from the TS, the AEP and the POA, and were in continuity with this structure (Fig. 3a–d,i,j,m,n, Fig. 7a,b,e,f and Supplementary Fig. 5). In *Nkx2.1-Cre*^*+*^*/Rosa-DTA* brains, the number of Nkx2.1-derived Nestin^+^ or GLAST^+^ astrocytes in the AC midline and in the ‘tunnel-like' astroglial structure surrounding the AC were reduced from E14.5 to E18.5 (in E18.5, only 28.73% of GLAST^+^/Olig2^−^ and 33.56% of GLAST^+^/Olig2^+^ remaining astrocytes; *P*<0.01, *n*=5, Student's *t*-test; Fig. 3e,f,k,l,o–r). Moreover, some GLAST^+^/GFAP^+^ astrocytes that were still left at the AC midline were completely disorganized (Figs 3f,g,p and 7c,d and Supplementary Fig. 7d–f). Hence, the cell ablation strategy successfully leads to a significant reduction of Nkx2.1-derived neurons and astrocytes in the AC region.

Thereafter, we investigated the effect of the loss of Nkx2.1-derived cells on the AC development by using the control *Nkx2.1-Cre*^*−*^*/Rosa-DTA* and mutant *Nkx2.1-Cre*^*+*^*/Rosa-DTA* embryonic brains beginning E14.5. At E14.5, the AC axons labelled with the axonal marker, L1 (L1 cell adhesion molecule) displayed similar trajectories in control *Nkx2.1-Cre*^*−*^*/Rosa-DTA* and mutant *Nkx2.1-Cre*^*+*^*/Rosa-DTA* brains (Fig. 5e–h). The axons successfully approached the midline in both control and mutant brains. However, the axons in mutant brains appeared to grow slowly and were slightly defasciculated at the midline compared with control axons of the AC that were tightly bundled together. At E15.5, the developing axons of the AC in mutant brains were observed to deviate from their normal path just before reaching the AC midline centre (Fig. 5k,l) compared with control littermates where a characteristic straight and compact AC structure was seen (Fig. 5i,j). In mutant brains, the AC was divided into several loosely bundled tracts with diverging orientation. Similar results were obtained from E16.5 to E18.5 where the AC was also not formed properly and axons were distracted from the customary path in mutant brains (Figs 5o,p and 7c,d,g,h,k,l and Supplementary Fig. 8b,d) compared with controls (Figs 5m,n and 7a,b,e,f,i,j and Supplementary Fig. 8a,c). Although AC axons remained close to the midline, they did not cross it and primarily separated into two or three significantly disoriented tracts remaining within either ipsilateral side of it. One of the major tracts was deflected rostro-dorsally towards the TS and the hippocampus, and the other two tracts were misrouted medially and ventrally. Sagittal sections confirmed the absence of a proper AC in the mutant brains compared with control brains (Supplementary Fig. 7). In addition, the disoriented axons of the AC were observed to be significantly defasciculated compared with control brains. The deflected axons projected rostro-dorsally towards the SEP and intermixed with the hippocampal axons that project to the fornix. Moreover, the hippocampal axons that project to the fornix were also affected and did not develop properly in mutant brains (Fig. 7g,h and Supplementary Fig. 7d–f).

Thereafter, we performed neuroanatomical tract tracing analysis to confirm the origin of the trajectory of the axonal tracts of the AC in mutant *Nkx2.1-Cre*^*+*^*/Rosa-DTA* brains (Fig. 7q–s). The crystals of DiI (1,1′-dioctadecyl 3,3,3′,3′-tetramethylindocarbocyanine perchlorate) were placed in the two lateral sides of the AC. Results revealed deviated trajectories of the AC axons (Fig. 7q–s). Instead of projecting towards the AC midline centre, the axonal trajectories were either stalled before reaching the midline or misrouted dorsally towards the TS and F (Fig. 7q–s). These results are in accordance with the observations made in coronal and sagittal sections of the mutant brains compared with control brains. Hence, ablation of Nkx2.1-derived cells leads to deflection of axons of AC from the normal path and improper development of the AC at the midline centre.

Next, to unravel the extent of defects due to distinct neuronal or glial ablation, neuron-specific enolase (NSE)-stop-DTA *(NSE-DTA)* mice^41^ were crossed with the *Nkx2.1-Cre* mice, which resulted in the specific ablation of the *Nkx2.1*-derived neurons only. Compared with the *Nkx2.1-Cre*^*+*^*/Rosa-DTA:GAD67-GFP* mice, the *Nkx2.1-Cre*^*+*^*/NSE-DTA:GAD67-GFP* mice displayed similar reductions of GAD67-GFP^+^ neurons, with a loss of 63.83±6.73% neurons at the AC midline (Fig. 6f,i). To analyse the effects of these two different cell ablation strategies on axonal guidance, we performed immunostaining with the axonal marker L1 on *Nkx2.1-Cre*^*+*^*/NSE-DTA:GAD67-GFP* mice and compared the results with previous observations made in the *Nkx2.1-Cre*^*+*^*/Rosa-DTA:GAD67-GFP* mice (Figs 6e and 7m,n). Interestingly, in *Nkx2.1-Cre*^*+*^*/NSE-DTA:GAD67-GFP* mice, the axonal guidance defects were weaker than in *Nkx2.1-Cre*^*+*^*/Rosa-DTA:GAD67-GFP* mice. In *Nkx2.1-Cre*^*+*^*/NSE-DTA:GAD67-GFP* mice, the axons of the AC exhibited different types of defects: (1) in some cases, they were able to cross the midline but the AC tract was thinner by ∼30% in *Nkx2.1-Cre*^*+*^*/NSE-DTA:GAD67-GFP* mice compared with controls (Fig. 6e,f,j); (2) in other cases, they did not cross the midline and were misrouted towards the TS where they intermixed with the axons of the fornix (Fig. 7m,n). Consequently, these results indicated that the *Nkx2.1*-derived GABAergic neurons played a role in the guidance of the AC axons. Since the defects were stronger in *Nkx2.1-Cre*^*+*^*/Rosa-DTA:GAD67-GFP* when glia and neurons were depleted than, in *Nkx2.1-Cre*^*+*^*/NSE-DTA:GAD67-GFP* mice, where only neurons were ablated, it can be deduced that all cells act in synergy, and that the Nkx2.1^+^ glia have a major role in axonal guidance in the AC.

Since Nkx2.1 regulates the specification of two different subclasses of glial cells, astrocytes and polydendrocytes, we use the elimination strategy to determine whether the loss of one or both glial populations contributes toward the guidance defects in *Nkx2.1-Cre*^*+*^*/Rosa-DTA* mutant brains. For this, *NG2-Cre* mice were crossed with *Rosa-DTA* mice to selectively deplete the NG2^+^ polydendrocytes. Analysis of *NG2-Cre*^*+*^*/Rosa-DTA:GAD67-GFP* mice verified that GAD67-GFP^+^ neurons and astrocytes were normal in the AC, and no axonal guidance defects were observed within the commissure (Figs 6g–i and 7o,p). This implies that the loss of astrocytes accounts for the significant guidance defects found in the *Nkx2.1-Cre*^*+*^*/Rosa-DTA* mutant brains.

Altogether, these results demonstrate that *Nkx2.1*-derived GABAergic neurons and astrocytes synergistically cooperate to guide axons across the AC and help them reach their respective targets during embryonic development.

### Slit2 is required by Nkx2.1-derived cells to guide AC axons

**Figure 8:**
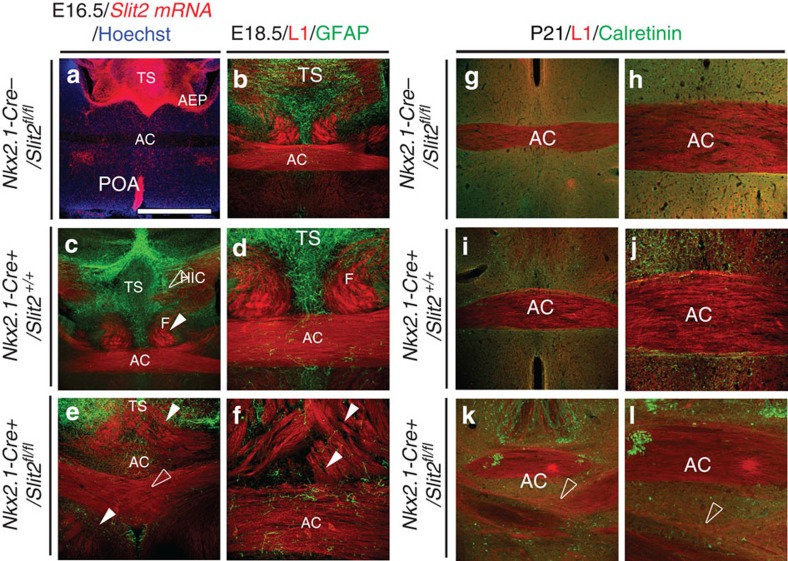
Midline guidance defects of the AC axons in *Nkx2.1-Cre*^*+*^*/Slit2*^*fl/fl*^ mice. (**a**) *In situ* hybridization for *Slit2* mRNA on AC coronal slices from control mice at E16.5 (*n*=3). The three regions, namely, the TS, the AEP and the POA, that harbour Nkx2.1-regulated precursors and glia, displayed strong expression of the repulsive molecule *Slit2*. (**b**–**l**) Double immunohistochemistry for L1 and GFAP (**b**–**f**), and L1 and Calretinin (**g**–**l**) on coronal sections from control *Nkx2.1-Cre*^*−*^*/Slit2*^*fl/fl*^ (*n*=3; **b**,**g**,**h**), control *Nkx2.1-Cre*^*+*^*/Slit2*^*+/+*^ (*n*=3; **c**,**d**,**i**,**j**) and mutant *Nkx2.1-Cre*^*+*^*/Slit2*^*fl/fl*^ (*n*=6; **e**,**f**,**k**,**l**) mice at E18.5 (**b**–**f**) and at P21 (**g**–**l**). In control *Nkx2.1-Cre*^*−*^*/Slit2*^*fl/fl*^ and *Nkx2.1-Cre*^*+*^*/Slit2*^*+/+*^ mutant mice (**a**–**d**,**g**–**j**), a normal AC was observed in E18.5 and P21 coronal sections. On the other hand, the L1^+^ commissural axons in mutant *Nkx2.1-Cre*^*+*^*/Slit2*^*fl/fl*^ mice displayed highly aberrant projection patterns (**e**,**f**,**k**,**l**). At both ages, E18.5 and P21, the axons were observed to criss-cross at the midline (open arrowhead, **e**,**k**,**l**). At E18.5, some of the axons projected dorsally invading the TS area while some others projected ventrally invading the POA area (solid arrowhead, **e**,**f**). Midline GFAP^+^ astrocytes were reduced and mispositioned in and around the AC in *Nkx2.1-Cre*^*+*^*/Slit2*^*fl/fl*^ mice (**e**,**f**) when compared with control (**b**–**d**). HIC, hippocampal commissure. Cell nuclei were counterstained in blue with Hoechst (**a**). The scale bar shown in panel **a** corresponds to the following length for panel(s) specified in brackets: 675 μm (**a**–**c**,**e**,**g**,**i**,**k**); 320 μm (**d**,**f**,**h**,**j**,**l**).

In order to identify the guidance cues produced by *Nkx2.1*-derived astroglia and neurons that guide AC axons, we performed *in situ* hybridization studies. Slit2, through Robo receptors, has been previously shown to repel many developing forebrain tracts^42^. Moreover, Slit2 and Robo1/2 mutants exhibit abnormal development of axonal tracts at the level of AC^5^,^6^,^8^. *In situ* hybridization for *Slit2* mRNA in control *Nkx2.1-Cre*^*−*^*/Rosa-DTA* and *Nkx2.1-Cre*^*−*^*/Slit2*^*fl/fl*^ coronal slices at E16.5 revealed that the *Nkx2.1*-regulated precursors and surrounding cells of the subpallium, including the TS, the AEP and the POA, strongly expressed *Slit2* (Fig. 8a and Supplementary Fig. 9a,b). By contrast, in *Nkx2.1-Cre*^*+*^*/Rosa-DTA* mice and *Nkx2.1-Cre*^*+*^*/Slit2*^*fl/fl*^, we could observe that the expression of *Slit2* in Nkx2.1^+^ cells was reduced in the POA and the TS compared with the control brains at E16.5 (Supplementary Fig. 9c–f).

To test the potential involvement of Slit2 in the guiding function of Nkx2.1^+^ cells on developing commissural axons, we examined the AC of *Nkx2.1-Cre*^*+*^*/Slit2*^*fl/fl*^ mutant mice compared with controls (*Nkx2.1-Cre*^*−*^*/Slit2*^*fl/fl*^ and *Nkx2.1-Cre*^*+*^*/Slit2*^*+/+*^) and *Nkx2.1-Cre*^*+*^*/Rosa-DTA* mice (Figs 7 and 8). The AC in *Slit1*^*−/−*^ mice was identical to *Slit1*^*+/+*^ possibly because Slit2 is still present in these mice as shown by previous studies in *Slit1*^−/−^ mutants^6^. Immunohistochemistry for GFAP at E18.5 indicated that GFAP^+^ astrocytes surrounding the AC were similarly displaced in *Nkx2.1-Cre*^*+*^*/Slit2*^*fl/fl*^ mice and in *Nkx2.1-Cre*^*+*^*/Rosa-DTA* mice (Figs 7c,d and 8e,f). *Nkx2.1-Cre*^*+*^*/Slit2*^*fl/fl*^ mice exhibited AC defects as severe as in *Nkx2.1-Cre*^*+*^*/Rosa-DTA* mice (Figs 7c,d,g,h,k,l and 8e,f). At E18.5, the AC tract appeared to be tripartitioned into dorsally and ventrally projecting commissural axons, while some others that crossed the midline through the normal AC path displayed an abnormal crossing pattern (Fig. 8e,f). The aberrant bifurcation of dorsal and ventral AC axons that failed to cross the midline was also observed previously in *Nkx2.1-Cre*^*+*^*/Rosa-DTA* mice. However, all commissural axons in *Nkx2.1-Cre*^*+*^*/Rosa-DTA* mice failed to cross the midline unlike in *Nkx2.1-Cre*^*+*^*/Slit2*^*fl/fl*^ mice. This suggests that in *Nkx2.1-Cre*^*+*^*/Slit2*^*fl/fl*^ mice, AC axons were not restricted to a defined tract as in control mice. *Nkx2.1-Cre*^*+*^*/Slit2*^*fl/fl*^ mice survived postnatally, and AC defects were still observed at postnatal age P21 (Fig. 8k,l). Although, the dorsally and ventrally projecting AC axons were not seen at postnatal ages, the enlarged ectopic AC with axonal crossing defects at the midline persisted.

Consequently, these results indicate that expression of the repellent molecule Slit2 in *Nkx2.1*-derived cells of the TS and the AEP/POA is participating in the guidance of AC axons.

## Discussion

Our results, altogether, show that during embryonic development midline glial cells present at the AC midline originate from the ventral telencephalon and play an important role during AC development. While the *Nkx2.1* homeobox gene is known to be required for the specification of GABAergic interneurons in the ventral telencephalon, we have found that, in addition, it regulates glial (astrocytes and polydendrocytes) differentiation. The *Nkx2.1*-derived cells, GABAergic interneurons and astrocytes, play a key role in the guidance and formation of the AC commissural tract in the ventral telencephalon. The depletion of either of the *Nkx2.1*-derived cells, or more severely of all, perturbs the development of the AC. The analysis of *Nkx2.1-Cre*^*+*^*/Slit2*^*fl/fl*^ mice reveals a novel and essential role of Slit2 in the function of Nkx2.1-derived guidepost cells in the pathfinding of AC axons. The present study, therefore, gives new insights into the mechanisms involved in axon guidance in the AC and indicates that Nkx2.1-derived neurons work in conjunction with their astroglial partners to guide commissural axons of the AC.

It has been documented that the neural development takes place in a specific sequence, wherein neurogenesis is followed by gliogenesis^28^,^29^,^30^,^31^,^32^,^33^,^39^,^43^. It is believed that the neurons are produced during the embryonic stages (from E11 onwards), followed by oligodendrocytes and then astroglia that are generated at the end of neurogenesis (after E17). However, on the other hand, one study has also reported early appearance (between E13 and P2 with a peak at E14) of astrocytes in the embryonic CC^34^. Hence, a detailed analysis of the embryonic astroglia that occupy the embryonic brain is still required.

The transition to astrogliogenesis depends upon the intercommunication (cooperative and/or inhibitory) between regulatory transcription factors whose expression and activity is, in turn, controlled by different signalling pathways, but still the exact underlying mechanisms are largely unknown^28^,^44^. Our studies have advanced the characterization of an astrogliogenesis phase that takes place during the course of late neurogenesis in the embryonic central nervous system (CNS). Using labelling for Ki67 and cell-specific markers, BrdU birth dating and inducible mice, we found that a multitude of astrocytes were generated at embryonic stages from E14.5 to E16.5. Hence, our study provides additional evidence that gliogenesis overlaps with late neurogenesis in the embryonic brain, and gives precise information concerning the time of appearance of astroglia in the embryonic AC.

The transcription factor Nkx2.1 is important for the proper specification of the ventral telencephalic area, the MGE and the AEP/POA, and is expressed in the subpallial progenitors that occupy these regions^19^,^23^,^24^,^25^,^45^. The combination of Nkx2.1 staining together with the astroglial markers confirmed the presence of several Nkx2.1^+^ precursor cells at the germinal sites in both the subpallial domains, the MGE and the POA. In addition, migratory streams comprised of Nkx2.1^+^ post-mitotic astroglial cell populations exited the ventricular and the SVZs. Surprisingly, a third region localized in the SEP, the TS, contained several Nkx2.1^+^ precursors that were also positive for the astroglial markers. It localized directly ventral to the hippocampal commissure and dorsal to the AC. *Ex vivo* and *in utero* electroporation tracing studies confirmed that the three germinal zones, MGE, POA and TS, could indeed give rise to Nestin^+^, GLAST^+^ and GFAP^+^ post-mitotic astrocyte-like cells. Further investigations involving faithful cell lineage tracing with time-resolved permanent labelling of newly generated Nkx2.1-derived cells could be done to further examine the precise origin of AC Nkx2.1-derived astrocytes. It has been shown that in the absence of Nkx2.1, the progenitors of the MGE and the AEP are re-specified to a more dorsal fate and are comparable to LGE progenitors^20^. This re-specification leads to drastic loss of GABAergic interneurons in the neocortex^17^,^20^,^21^. In our study, we performed selective ablation of *Nkx2.1*-derived post-mitotic cells with the aid of *Nkx2.1-Cre*^*+*^/*Rosa-DTA* embryos. The precursors were left unaffected due to a delay of action of the diphtheria toxin. Upon investigation with several glial markers, we observed that in *Nkx2.1-Cre*^*+*^/*Rosa-DTA* mice, a drastic loss of astrocytes occurred at the AC midline and surrounding tunnel region. Finally, our lineage-tracing studies using *Nkx2.1-Cre*^*+*^/*Rosa-YFP* mice revealed that the *Nkx2.1*-derived astrocytes can be divided into the following two major categories: astrocyte-like glial cells that are GLAST^+^/GFAP^+^/Olig2^−^ and those that are GLAST^+^/GFAP^−^/Olig2^+^ (Supplementary Fig. 2). Altogether, these results reveal that a diverse population of astrocytes is generated from Nkx2.1^+^ progenitors within the ventral telencephalon.

The role of guidepost neurons within the CC has already been brought forward through our previous studies^12^,^14^,^15^,^16^. The astroglia, situated in the regions surrounding the CC^2^,^4^,^7^,^9^,^10^,^11^,^46^, and even polydendrocytes^47^ have been implicated in guiding callosal axons. Yet, there is no functional data available that displays the definitive role of glia in guiding commissural axons through the AC midline. As also previously reported^20^, we observed that the AC failed to form in the embryos lacking Nkx2.1. It is then possible that either or both the populations of GABAergic neurons and/or astroglia could be responsible for the axonal defects seen in *Nkx2.1*^*−/−*^ mice brains. Hence, to delineate the specific involvement of each population separately, we made use of a selective cell ablation strategy. The *Nkx2.1-Cre*^*+*^*/Rosa-DTA* mice were used to ablate all the Nkx2.1-derived cells, including neurons and glia, while *Nkx2.1-Cre*^*+*^*/NSE-DTA* mice produced specific ablation of only the Nkx2.1-derived neuronal cells. Both of these mice have the added advantage over *Nkx2.1*^*−/−*^ mice that precursors were not affected and the MGE formed normally. In *Nkx2.1-Cre*^*+*^*/Rosa-DTA* mice, owing to the time of expression and efficiency of the toxin, the ventral telencephalon progenitors were not ablated, but the glia and GABAergic neurons at the midline within the AC and the ‘tunnel-like' astroglia forming the palisade bordering the AC were reduced. This cellular loss was accompanied by major defects at the AC. As early as E14.5, although the axons of the AC in mutant brains approached the midline similarly to those in control brains, evident defasciculation of the AC axons was visualized in mutant *Nkx2.1-Cre*^*+*^*/Rosa-DTA* mice. The defects were manifest already by E15.5 wherein the axons contributing to the AC tract were largely disoriented in the mutant brains compared with control brains of same age. Similarly, at E16.5 and E18.5, the AC axons reach the midline, but failed to cross the midline and were segregated into two or three major ipsilateral tracts. One of the tracts was deflected dorsally towards the TS and the hippocampal region, whereas the other two stopped abruptly before reaching the midline and appeared to be misrouted either medially or ventrally. A clear correlation could be drawn between the depletion of Nkx2.1-derived cells and severe developmental defects of the AC. Through these results, it was obvious that the Nkx2.1-derived cells occupy the AC midline centre region even before the arrival of the commissural axons and aid to the guidance of the growing axonal tracts at the AC midline centre.

Next, we aimed to delineate the contribution of the Nkx2.1-derived neurons and glia towards the development of the AC. Analysis of *Nkx2.1-Cre*^*+*^*/NSE-DTA* mice displayed a severe loss of GABAergic neurons comparable to the *Nkx2.1-Cre*^*+*^*/Rosa-DTA* mice. Interestingly, these mice also exhibited defects in the axonal guidance, but they were less severe than those of *Nkx2.1-Cre*^*+*^*/Rosa-DTA* mice. In some cases, the axons of the AC were misrouted from their normal path and began to progress towards the TS, and intermixed with axons of the fornix. In some other cases, the axons adopted normal conformation and crossed the midline, but the AC tract was significantly narrower than the WT AC tract. The defects visualized by the loss of neurons only were not as drastic as those observed after the loss of both neurons and glia. Taken together, these results display the synergistic cooperation between the Nkx2.1-derived GABAergic neurons and glia towards the accurate formation of the AC. Whereas, *NG2-Cre*^*+*^*/Rosa-DTA:GAD67-GFP* mice did not display any significant defects, thus, excluding the possibility of involvement of polydendrocytes in these guidance defects. Therefore, based on their respective localization and molecular signature, it is likely that the Nkx2.1-derived neurons and astrocytes contribute to guidance mechanisms in the embryonic brain. Future studies aimed towards further elucidating the function of Nkx2.1-derived astroglial populations solely would improve our understanding of the guidance mechanisms underlying AC formation.

The *Nkx2.1*-regulated precursors and the surrounding post-mitotic cells of the subpallium, including the TS, the AEP and the POA, showed strong expression of the repellant molecule *Slit2* in WT mice. This probably confines the AC tract in a Slit2^−^ permissive path surrounded by *Slit2*^+^ cells. Previous studies have highlighted the importance of Slit2 towards proper AC formation^6^. However, until now, the identity of these *Slit2* expressing cells was not known. By using a diphtheria toxin-based cell ablation strategy in *Nkx2.1-Cre*^*+*^*/Rosa-DTA* mice and a conditional gene inactivation approach in *Nkx2.1-Cre*^*+*^*/Slit2*^*fl/fl*^ mice at E16.5, we observed that the expression of Slit2 was reduced in Nkx2.1-derived cells. At E18.5, the AC tract of the *Nkx2.1-Cre*^*+*^*/Slit2*^*fl/fl*^ mutant mice appeared to be tripartitioned into dorsally projecting and ventrally projecting commissural axons, and into some others that crossed the midline displaying a criss-crossing pattern. Interestingly, the tripartitioned AC phenotype exhibited by *Nkx2.1-Cre*^*+*^*/Slit2*^*fl/fl*^ mutant mice is similar to the previously described *Slit*1^−/−^;*Slit2*^−/−^ double mutants^6^. The aberrant criss-crossing pattern in *Nkx2.1-Cre*^*+*^*/Slit2*^*fl/fl*^ appears to have occurred due to axons, originating from both hemispheric sides, which cross the midline and continue to extend away from it and hence, visually appear as criss-crossing. It could also be due to back and forth growth of axons as shown in *Slit1*^−/−^;*Slit*2^−/−^ double mutants^6^. In addition, some of the misdirected axons observed at the midline in *Nkx2.1-Cre*^*+*^*/Slit2*^*fl/fl*^ mutant mice could be neocortical in origin^6^.

The aberrant bifurcation of dorsally and ventrally projecting commissural axons was observed previously in our *Nkx2.1-Cre*^*+*^*/Rosa-DTA* mice too. However, the phenotype resulting from the absence of *Slit2* in *Nkx2.1-Cre*^*+*^*/Slit2*^*fl/fl*^ mice is comparatively milder when compared with the one observed upon reduction of Nkx2.1-derived cells in *Nkx2.1-Cre*^*+*^*/Rosa-DTA* mice. This difference observed between the two mouse mutant lines could be owing to the fact that in former mice the expression of DTA toxin leads to depletion of Nkx2.1-derived cells at the midline, which are indispensable for the proper formation of the AC, while in the later *Nkx2.1-Cre*^*+*^*/Slit2*^*fl/fl*^ mice, only expression of Slit2 is depleted in Nkx2.1-derived cells, which still continue to exist. Evidently, the depletion of repellent cue Slit2 leads to projection defects demonstrated by the criss-crossing of the commissural axons at the midline, and aberrant dorsal and ventral projections deviated from the normal path. The defects observed in embryonic *Nkx2.1-Cre*^*+*^*/Slit2*^*fl/fl*^ mice continued to exist postnatally, and defective AC and mispositioned glia are observed in adult *Nkx2.1-Cre*^*+*^*/Slit2*^*fl/fl*^ mice, too. Thus, these findings underline the potential involvement of Slit2 in the guidance function of Nkx2.1-derived cells on developing commissural axons. The mode of Slit2 action could be either direct at the level of commissural axons since they are known to express the Robo1/2 receptors for Slit2 or indirect via the mispositioning of the guidepost cells.

*Slits* have been previously shown to be a repulsive axon guidance cue and to play a role during commissural axon crossing at the CC and AC^5^,^6^,^8^,^10^,^11^,^16^. Our current analysis displays compelling and additional evidence about the role of *Slit2* during AC development. We found that the action of *Slit2* is mediated via the specific Nkx2.1-derived cell populations that regulate the formation of the AC. This regulation could occur at several levels, which definitely includes both spatial and temporal aspects. We show that the timing of the generation of the Nkx2.1-derived cells precedes the arrival of the commissural axons at the midline centre. The Nkx2.1-derived cells through Slit2 action provide guidance cue to the axonal tracts that restrain them into the typical compact pathway seen in control animals. Although we know now that the Nkx2.1-derived midline glial and neuronal populations use *Slit2* to guide the axons through the commissure, we cannot exclude the expression of other molecules in Nkx2.1-derived cells since the phenotype resulting from their depletion in *Nkx2.1-Cre*^*+*^*/Rosa-DTA* brains is stronger than that observed by absence of Slit2 alone in Nkx2.1-derived cells. *Sema3B* has been shown to be important for the correct positioning of the AC^48^. Interestingly, its expression overlaps with that of *Slit2* found in our analysis. Thus, this raises the possibility that *Sema3B* and *Slit2* might act through a concerted action. Furthermore, absence of semaphorin receptor neuropilin-2 leads to a complete absence of AC^49^,^50^. Similar defects were visualized on inactivation of the *neuropilin-2* ligand *Sema3F* (ref. 51). Moreover, Eph family members have also been implicated towards proper development of the AC. For example, *Nuk* plays an important role towards pathfinding of axons required for AC formation^52^, and combinatorial action of EphB2 and EphA4 receptors is required for proper guidance of axonal tracts forming the AC^53^. *Draxin* mutants also display abnormal development of AC^54^. Interestingly, deletion of either of the aforementioned molecules leads to defects where the AC axons never approach the midline and are completely stalled in the lateral part of the telencephalon. However, our study reveals that axonal defects observed in conditional mutants lacking Nkx2.1-derived cells—glia and neurons—are centred at the midline proper. Interestingly, phenotypically similar defects wherein the axonal tracts are disoriented towards rostral or caudal areas have been seen with mutants lacking allelic variations of polysialyltransferases^55^ or those lacking JNK/stress-activated protein kinase-associated protein 1 (ref. 56). Future studies focused towards identification of additional molecules that could be expressed by the Nkx2.1-derived cells, or/and the precise mode of action of Slit2, can provide more information about the AC formation at the midline.

These findings suggest that, with the aid of Nkx2.1, further analysis into the regulation and developmental genetics of astrogliogenesis can undoubtedly improve our knowledge about a plethora of neurological disorders due to improper glia generation in the brain^57^,^58^,^59^. In conclusion, this study brings a number of new identified genetic partners involved in the regulation of commissures formation. It consolidates our understanding of the molecular and cellular mechanisms activated in this process. It also adds to the genetic evaluation of the multiple forms of commissural defects identified in the human brain.

## Methods

### Animals

All studies on mice of either sex have been performed in compliance with the national and international guidelines. For staging of embryos, midday of the day of vaginal plug formation was considered as embryonic day 0.5 (E0.5). WT mice maintained in a C57Bl/6 genetic background were used for developmental analysis of the AC. We used heterozygous *GAD67*^*_*^*GFP* knock-in mice, described in this work as *GAD67-GFP* mice^60^. GAD67^_^GFP embryos could be recognized by their GFP fluorescence. PCR genotyping of these lines was performed as described previously^14^. We used *Nkx2.1*-*Cre*^61^, *GLAST-Cre:ERT2* (The Jackson Laboratory, Bar Harbor, Maine, USA, Tg(Slc1a3-cre/ERT)1Nat/J), *Olig2-Cre ERT*^*TM*^^62^ and *NG2-Cre* (The Jackson Laboratory: B6;FVB-Tg(Cspg4-cre)1Akik/J)^63^ transgenic mice that have been described previously. The control *GLAST-Cre:ERT2*^*+*^*/Rosa26-YFP* brains did not show any YFP labelling without tamoxifen treatment. The reporter *Rosa26R–YFP*^64^ mouse line was used to reliably express YFP under the control of the Rosa26 promoter upon Cre-mediated recombination. The reporter Rosa26:lacZ/DTA (Rosa-DTA) mouse line^40^ was used to conditionally express the cytototoxic diphtheria toxin polypeptide toxic fragment A (DTA) allele under the control of ubiquitously active Rosa26 promoter. The NSE-stop-DTA (NSE-DTA) mice^41^ were used to induce the expression of highly potent DTA from NSE locus and resulted in specific ablation of neurons only. *Nkx2.1-Cre*^*+*^*/Slit2*^*fl/fl*^ mice were used to study whether Nkx2.1-derived cells may exert their guidance activity through Slit2 guidance factor. The *Slit2*^*flox*^ conditional allele containing loxP sequences flanking exon 8 was established at the Institut Clinique de la Souris, Illkirch, France; (http://www-mci.u-strasbg.fr). In the presence of Cre recombinase, this interrupts the Slit2 protein after amino acid Thr203 located in the first leucine-rich domain. For the induction of CreERT, tamoxifen (20 mg ml^−1^, Sigma, St Louis, MO) was dissolved at 37 °C in 5 ml corn oil (Sigma) pre-heated at 42 °C for 30 min. A single dose of 4 mg (250–300 μl) was administered to pregnant females by oral gavaging.

### *In utero* electroporation

To perform *in utero* electroporation, we adapted our previous protocol^65^. E14.5-timed pregnant *Nkx2.1*-Cre^+^/Rosa-YFP mice were anaesthetized with isoflurane (3.5% during induction and 2.5% during surgery) and embryos were exposed within the intact uterine wall after sectioning the abdominal wall. The DNA solution containing the expression vector encoding the red fluorescent protein (*pCAG-IRES-Tomato*; 2 mg ml^−1^) together with the dye Fast Green (0.3 mg ml^−1^; Sigma) was pressure injected focally (1–2 μl) into the lateral ventricle of embryos through a glass micropipette using a PicoSpritzer III (Parker Hannifin, Cleveland, OH). Each embryo, still in the uterus, was placed between tweezer-type electrodes (System CUY-650P5 Nepa Gene Co., Chiba, Japan) and was electroporated with five electrical square unipolar pulses (amplitude: 45 V; duration: 50 ms; intervals: 950 ms) powered by a BTX electroporator apparatus (model BTX ECM 830; Harvard Apparatus, Holliston, MA). By orienting the tweezer-type electrodes, we were able to preferentially electroporate ventral pallium precursors that give rise in part to embryonic glial cells. The embryos were quickly placed back into the abdominal cavity, and the muscular and skin body wall layers were sutured. The embryos were allowed to develop until E18.5.

### Slice cultures and *in vitro* focal electroporation

For E14.5–E16.5 telencephalic slice cultures, we used an *in vitro* model of telencephalic organotypic slices that has been previously described^14^. Embryos were dissected in ice-cold dissecting medium (MEM Gibco, ref. no. 11012-044 with 15 mM glucose and 10 mM Tris, pH 7–9). Brains were isolated and embedded in 3% low-melting point agarose (Invitrogen). Coronal sections (250 μm thick) were made using a vibratome and slices at the level of the AC, the TS, the MGE/LGE and the AEP/POA were collected in cold dissecting medium. Slices were cultured on nucleopore Track-Etch membrane (1 μm pore size; Whatman) in slice culture medium basal media eagle (BME)/hank's balanced salt solution (HBSS) (Invitrogen) supplemented with 1% glutamine, 5% horse serum and penicillin/streptomycin.

We applied an *in vitro* focal electroporation method using a Nepagene apparatus to transfect an expression vector into telencephalic slices. E14.5–E16.5 organotypic coronal slices from WT embryos were obtained as described above. The expression vectors encoding the green (*pCAG-IRES-EGFP)* or the red (*pCAG-IRES-Tomato*) fluorescent proteins were electroporated at a concentration of 1 mg ml^−1^ in sterile PBS 1 × (Sigma). Expression vectors were focally injected through a glass micropipette into the TS, the LGE, the MGE or the POA using a PicoSpritzer III (Parker Hannifin), and the slices were electroporated between platinum Petri dish and cover square platinum electrodes (System CUY-700-1 and CUY-700-2; Nepa Gene Co.) with two unipolar square pulses (amplitude: 100 V; duration: 5 ms; intervals: 500 ms) generated by a CUY21EDIT Electroporator (Nepa Gene Co.).

### Immunocytochemistry

Embryos were collected after caesarean section and quickly killed by decapitation. Their brains were dissected out and fixed by immersion overnight in a solution of 4% paraformaldehyde in 0.1 M phosphate buffer (pH 7.4) at 4 °C. Postnatal mice were profoundly anaesthetized and perfused with the same fixative and their brains post fixed for 4 h. Brains were cryoprotected in a solution of 30% sucrose in 0.1 M phosphate buffer (pH 7.4), frozen and cut in 50-μm-thick coronal sections or 50-μm-thick sagittal sections for immunostaining. For each immunostaining, we made use of several mice (between 4 and 10) for both control and mutant strains analysed.

Mouse monoclonal antibodies were as follows: BrdU (1/50; Monosan), Nestin (1/600; Pharmingen) and GFAP (1/500; Chemicon). Rat monoclonal antibody was L1 (1/200; Chemicon). Rabbit polyclonal antibodies were as follows: Calretinin (1/2,000; Swant), GFAP (1/500; DAKO), GFP (1/500; Molecular Probes, Eugene, OR), NG2 (1/100; Chemicon), Nkx2.1 (1/2,000; Biopat), Olig2 (1/500; Millipore), red fluorescent protein (RFP) (1/500; Labforce) and S100β (1/2,500; Swant). Guinea pig antibody was GLAST (1/200; Chemicon). Chicken antibody was GFP (1/500; Aves).

Fluorescence immunostaining was performed as follows: non-specific binding was blocked with 2% normal horse serum in PBS 1 × solution with 0.3% Triton X-100 for pre-incubation during permeabilization and incubations with antibodies. The primary antibodies were detected with Cy3-conjugated (Jackson ImmunoResearch Laboratories) and Alexa488-, Alexa594- or Alexa647-conjugated antibodies (Molecular Probes). Sections were counterstained with Hoechst 33258 (Molecular Probes), mounted on glass slides and covered in Mowiol 4–88 (Calbiochem, Bad Soden, Germany).

3,3′-diaminobenzidine (DAB) immunostaining was performed as follows: endogenous peroxidase reaction was quenched with 0.5% hydrogen peroxide in methanol and non-specific binding was blocked by adding 2% normal horse serum in Tris-buffered solutions containing 0.3% Triton X-100 for pre-incubation and incubations. The primary antibodies were detected with biotinylated secondary antibodies (Jackson ImmunoResearch Laboratories) and the Vector-Elite ABC kit (Vector Laboratories, Burlingame, CA). The slices were mounted on glass slides, dried, dehydrated and covered with Eukitt mounting medium.

### Axonal tracing

After overnight fixation in 4% paraformaldehyde (PFA) at 4 °C, fluorescent carbocyanide dye DiI crystal were placed in the lateral sides of the AC in 250-μm-thick vibratome coronal sections of E18.5 *Nkx2.1-Cre*^*+*^*/Rosa-DTA* mice (*n*=12; ref. 14). After 4 weeks at 37 °C in 4% PFA to allow dye diffusion, the samples were counterstained with Hoechst (Molecular Probes) and were imaged using a Leica MZ10F stereo microscope for fluorescence imaging and a SP5 Leica confocal microscope.

### BrdU-tracing studies

To label cells in the S-phase of the cell cycle at suitable embryonic stages (E12.5, E14.5 and E16.5), the pregnant female mice were injected intraperitoneally with a solution of 8 mg ml^−1^ of BrdU (Sigma) in PBS (0.15 M NaCl and 0.1 M phosphate buffer, pH=7.4) to a final concentration of 50 mg kg^−1^ body weight. To trace the date of genesis of the AC astrocytes, the WT and Nkx2.1-Cre^+^/Rosa-YFP pregnant females were killed when embryos were E18.5. The BrdU was revealed by fluorescence immunostaining after a treatment with 2 M HCl for 30 min at room temperature.

### Imaging

DAB-stained sections were imaged with a Zeiss Axioplan2 microscope equipped with × 10, × 20 or × 40 Plan neofluar objectives and coupled to a CCD camera (Axiocam MRc 1,388 × 1,040 pixels). Fluorescent-immunostained sections were imaged using confocal microscopes (Zeiss LSM 510 Meta, Leica SP5 or Zeiss LSM 710 Quasar) equipped with × 10, × 20 and × 40 oil Plan neofluar, and × 63 and × 100 oil Plan apochromat objectives. Fluorophore excitation and scanning were done with an Argon laser 458, 488 and 514 nm (blue excitation for GFP and Alexa488) with a HeNe laser 543 nm (green excitation for Alexa 594, CY3 and DiI), with a HeNe laser 633 nm (excitation for Alexa647 and CY5) and a diode laser 405 nm (for Hoechst-stained sections). Z-stacks of 10–15 planes were acquired for each AC coronal section in a multitrack mode avoiding crosstalk. Z-stacks of 10–20 sections were acquired for each AC section for the creation of isosurfaces with Imaris 7.2.1 software (Bitplane Inc.).

All three-dimensional (3D) Z stack reconstructions and image processing were performed with Imaris 7.2.1 software. To create real 3D data sets, we used the mode ‘Surpass'. Figures were processed in Adobe Photoshop CS4 and CS5, and schematic illustrations were produced using Adobe Illustrator CS4.

### Quantification of astroglial cell population of the AC

In 50-μm-thick brain sections of *Nkx2.1-Cre*^*+*^*/Rosa-YFP* embryos at E14.5 (*n*=3) and E16.5 (*n*=8), the astrocytes that were labelled for GLAST and the polydendrocytes that were labelled for NG2 were counted in the AC midline centre.

In 50-μm-thick brain sections of *Nkx2.1-Cre*^*−*^*/Rosa-DTA* (*n*=5) and *Nkx2.1-Cre*^*+*^*/Rosa-DTA* embryos (*n*=3) at E16.5, the astrocytes that were labelled for Nkx2.1 and GLAST were counted in the AC midline centre and the AC lateral sides from at least nine sections per AC.

In 50-μm-thick brain sections of *Nkx2.1-Cre*^*−*^*/Rosa-DTA* (*n*=5) and *Nkx2.1-Cre*^*+*^*/Rosa-DTA* embryos (*n*=5) at E18.5, the astrocytes that were labelled for Olig2 and GLAST were counted in the AC midline centre and the AC lateral sides from at least nine sections per AC.

The cell densities were reported per volume unit (number of cells per mm^3^). The quantification was done using Imaris 7.2.1 software.

### Quantification of AC GABAergic neuronal population

In 50-μm coronal sections of Nkx2.1-Cre^−^/Rosa-DTA:GAD67-GFP (*n*=3), Nkx2.1-Cre^+^/Rosa-DTA:GAD67-GFP (*n*=4), Nkx2.1-Cre^−^/NSE-DTA:GAD67-GFP (*n*=6) and Nkx2.1-Cre^+^/NSE-DTA:GAD67-GFP (*n*=4), NG2-Cre^−^/Rosa-DTA:GAD67-GFP (*n*=3) and NG2-Cre^+^/ROSA-DTA:GAD67-GFP (*n*=3) embryos, GAD67-GFP^+^ GABAergic neurons of the AC were counted at E18.5 as the number of cells labelled for the GFP from 12 to 18 sections per AC. To study the density of GABAergic neurons, the values were quantified in each section stack and were reported per volume unit (number of cells per mm^3^). The quantifications were done using the spot mode analysis of Imaris 7.2.1 software. The cell densities were determined in two AC areas (medial and two lateral sides) and in the entire AC area (total).

### Statistical analysis

The results from all quantifications were analysed with the aid of Statview software (SAS Institute Inc.). For all analysis, values from at least three independent experiments were first tested for normality and the variance of independent populations were tested for equality. Values that followed a normal distribution were compared using Student's *t*-test. Values that did not follow a normal distribution were compared using Mann–Whitney and Kolmogorov–Smirnov nonparametric tests.

### Atlas and nomenclature

The neuroanatomical nomenclature is based on the ‘Atlas of the prenatal mouse brain'^66^.

## Author contributions

The experiments were conceived and designed by S.M., D.V., J.-P.H. and C.L. The experiments were performed by S.M., D.V. and C.L. S.M., D.V. and C.L. analysed the data. A.R.Y., H.F., Y.Y., O.M. and A.C. contributed reagents/materials/analysis tools. S.M., J.-P.H. and C.L. wrote the paper. All authors participated in the discussion of the data and in production of the final version of the manuscript.

## Additional information

**How to cite this article:** Minocha, S. *et al*. *Nkx2.1*-derived astrocytes and neurons together with Slit2 are indispensable for anterior commissure formation. *Nat. Commun*. 6:6887 doi: 10.1038/ncomms7887 (2015).

## Supplementary Material

Supplementary Information Supplementary Figures 1-9
