# Supplementary material for: Nkx2.1-derived astrocytes and neurons together with Slit2 are indispensable for anterior commissure formation
Source: Nat Commun. 2015 Apr 23;6:6887. doi: 10.1038/ncomms7887 (PMC4423212; doi:10.1038/ncomms7887)
Supplement: Supplementary Information — Supplementary Figures 1-9 [file ncomms7887-s1.pdf]

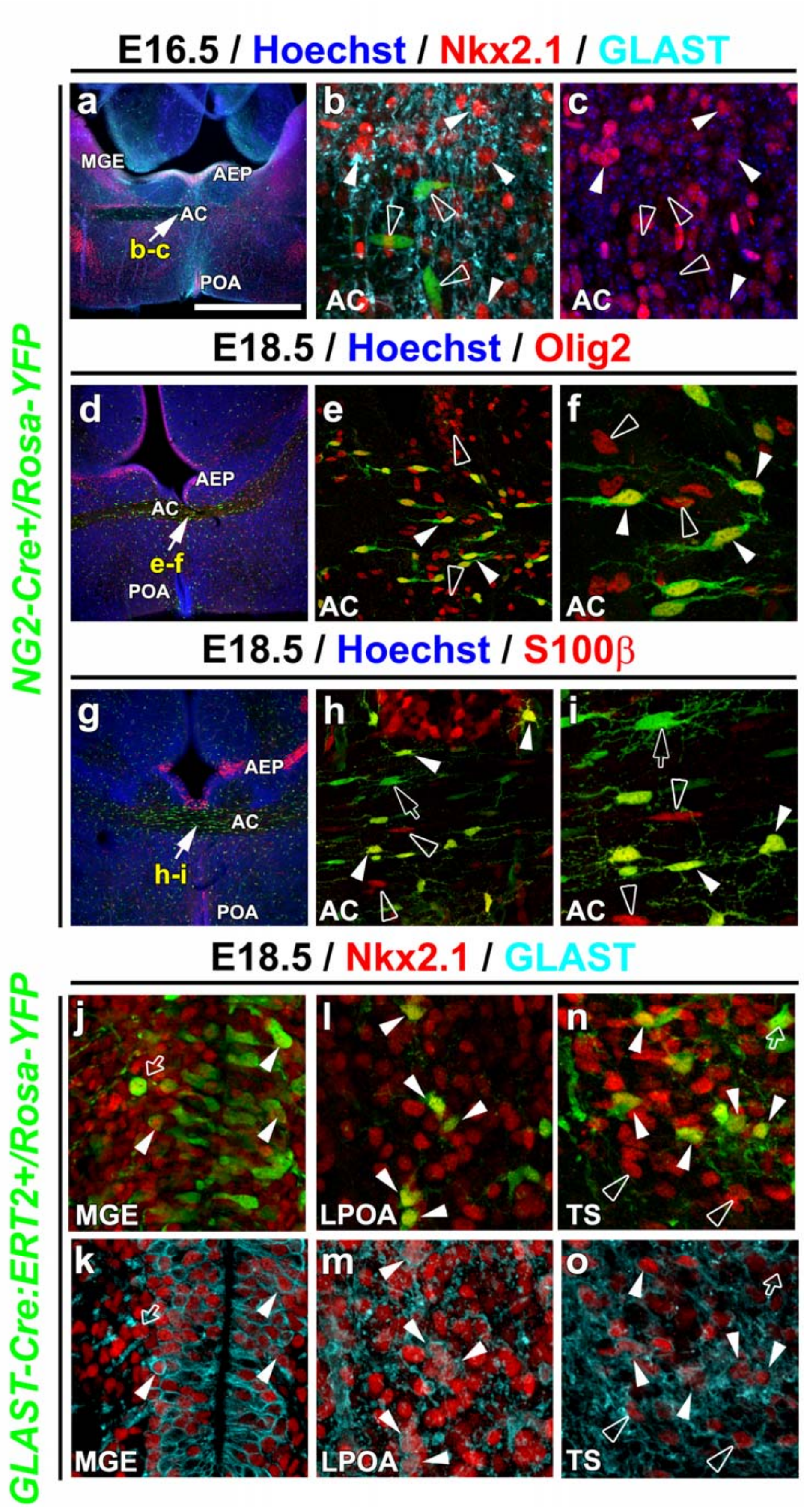

Supplementary Figure 1

Supplementary Figure 1. Presence of *Nkx2.1*-derived polydendrocytes within the embryonic AC.

(a-i) Triple immunohistochemistry for YFP, Nkx2.1 and GLAST (n=4) **(a-c)** and double immunohistochemistry for YFP and Olig2 (n=4) **(d-f)** and for YFP and S100β (n=3) **(g-i)** at E16.5-E18.5 on anterior commissure **(AC)** coronal sections from *NG2-Cre$^+$/Rosa-YFP* mice. **b-c, e-f,** and **h-i** are higher power views of the regions indicated by an arrow in **a, d,** and **g**, respectively. **(a-c)** At E16.5, while GLAST$^+$ astroglia of the AC expressed Nkx2.1 (solid arrowheads), Nkx2.1 was down-regulated in differentiated NG2-derived YFP$^+$ polydendrocytes (open arrowheads). **(d-f)** At E18.5, all the NG2-derived YFP$^+$ polydendrocytes within the CC and the AC co-expressed Olig2 (solid arrowheads), while not all Olig2$^+$ cells expressed NG2 (open arrowheads). **(g-i)** Many, though not all (open arrows), post-mitotic NG2$^+$ cells of the commissures were co-labeled by S100ß (solid arrowheads) and few S100β$^+$ glia of the AC did not express NG2 (open arrowheads).

(j-o) Triple immunohistochemistry for YFP, Nkx2.1 and GLAST at E18.5 on brain coronal sections from *GLAST-CreERT2$^+$/Rosa-YFP* mice (n=3). YFP$^+$/GLAST$^+$ astrocytes were observed to express Nkx2.1 in the VZ and the SVZ of the medial ganglionic eminence **(MGE) (j-k)**, the lateral part of the preoptic area **(LPOA) (l-m)** and the triangular septal nucleus **(TS) (n-o)** (solid arrowheads). Some GLAST$^+$ astroglia colabelled for Nkx2.1 did not express the YFP (open arrowheads in **n-o**). Some YFP+ cell colabelled for Nkx2.1 that did not express the GLAST may correspond to interneurons (open arrows in **j** and **n**).

Cell nuclei were counterstained in blue with Hoechst **(a, d** and **g)**. Colocalization between the green and the red channel was highlighted in yellow **(e-f**, **h-i, j, l** and **n)**. **(AEP)** anterior entopeduncular area, **(POA)** preoptic area. Bar = 675 µm in **a, d** and **g**; 100 µm in **e** and **h;** 45 µm in **b, c, j-o** and 40 µm in **f** and **i**.

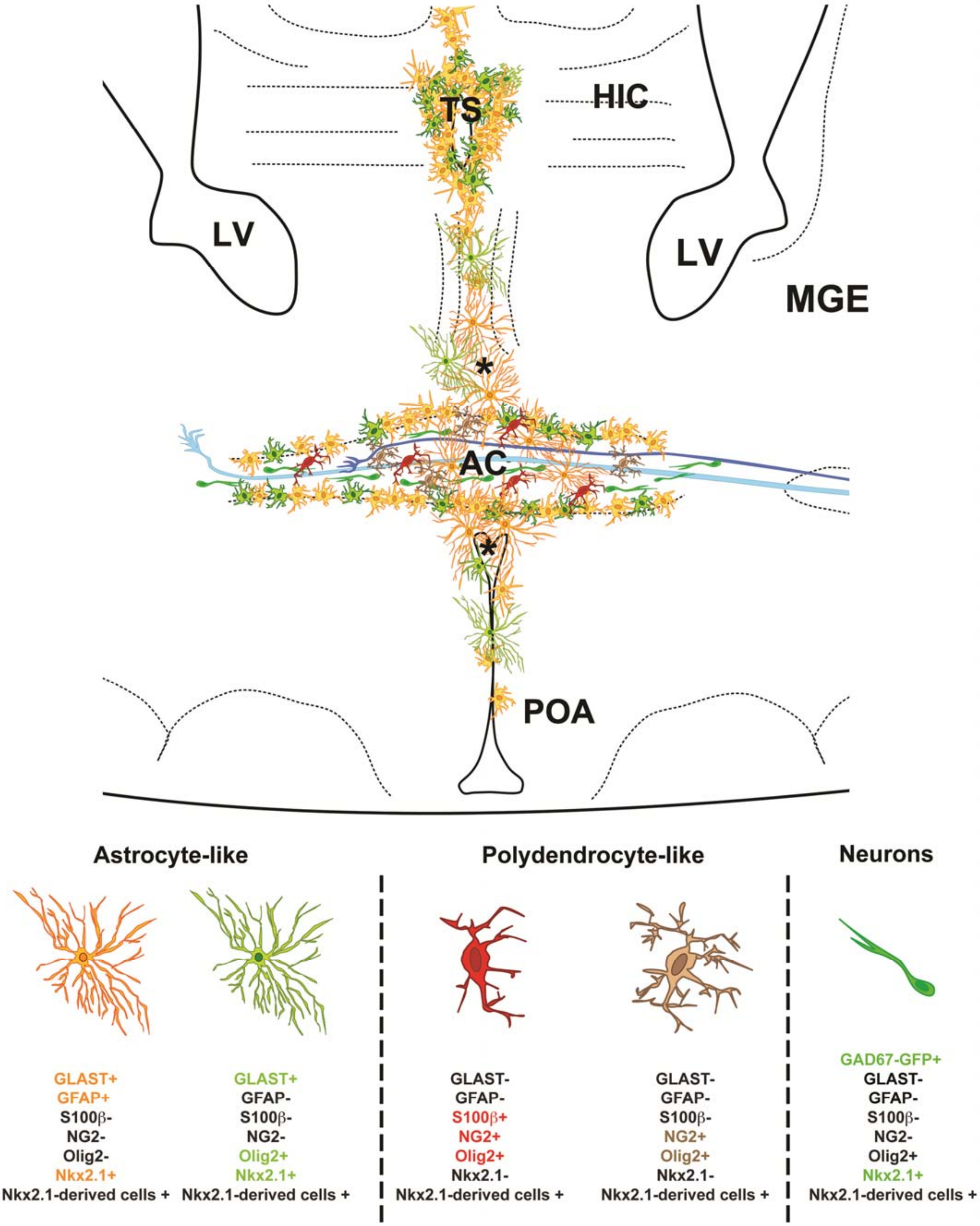

Supplementary Figure 2

Supplementary figure 2. Schematics showing four *Nkx2.1*-derived embryonic glial subtypes.

The schema represents a coronal view of the anterior commissure **(AC)** at E18.5, and summarizes the different types of *Nkx2.1*-derived glial and neuronal populations described in this study. The two types of astrocyte-like cell populations are shown, namely, the $GLAST^{+}/GFAP^{+}$ cells (yellow) and the $GLAST^{+}/Olig2^{+}$ cells (light green). Two types of polydendrocyte-like cells are shown, namely, the $NG2^{+}/Olig2^{+}/S100\beta^{+}$ cells (red), and the $NG2^{+}/Olig2^{+}/S100\beta^{-}$ cells (brown). GABAergic neurons are shown, namely, the GAD67-GFP^{-} cells (dark green). Under each cell-type category the expression profile of the different glial markers, employed to identify and characterize the glial cells used in combination with the Nkx2.1 antibody, is presented. $Nkx2.1^{-}$ polydendrocyte-like cells are derived from Nkx2.1 cells in the subpallium that loose the Nkx2.1 expression while leaving the progenitor zone. By contrast astrocyte-like cell populations still express Nkx2.1 until birth. The commissural axons are represented in blue and are seen to navigate through the AC glia and neurons.

The (+) sign indicates that the cell type was positively labeled by the listed marker, whereas the (–) sign indicates that the cell type was not labeled by the listed marker.

(HIC) hippocampal commissure, **(LV)** lateral ventricle, **(POA)** preotic area, **(MGE)** medial ganglionic eminence, **(TS)** triangular septal nucleus.

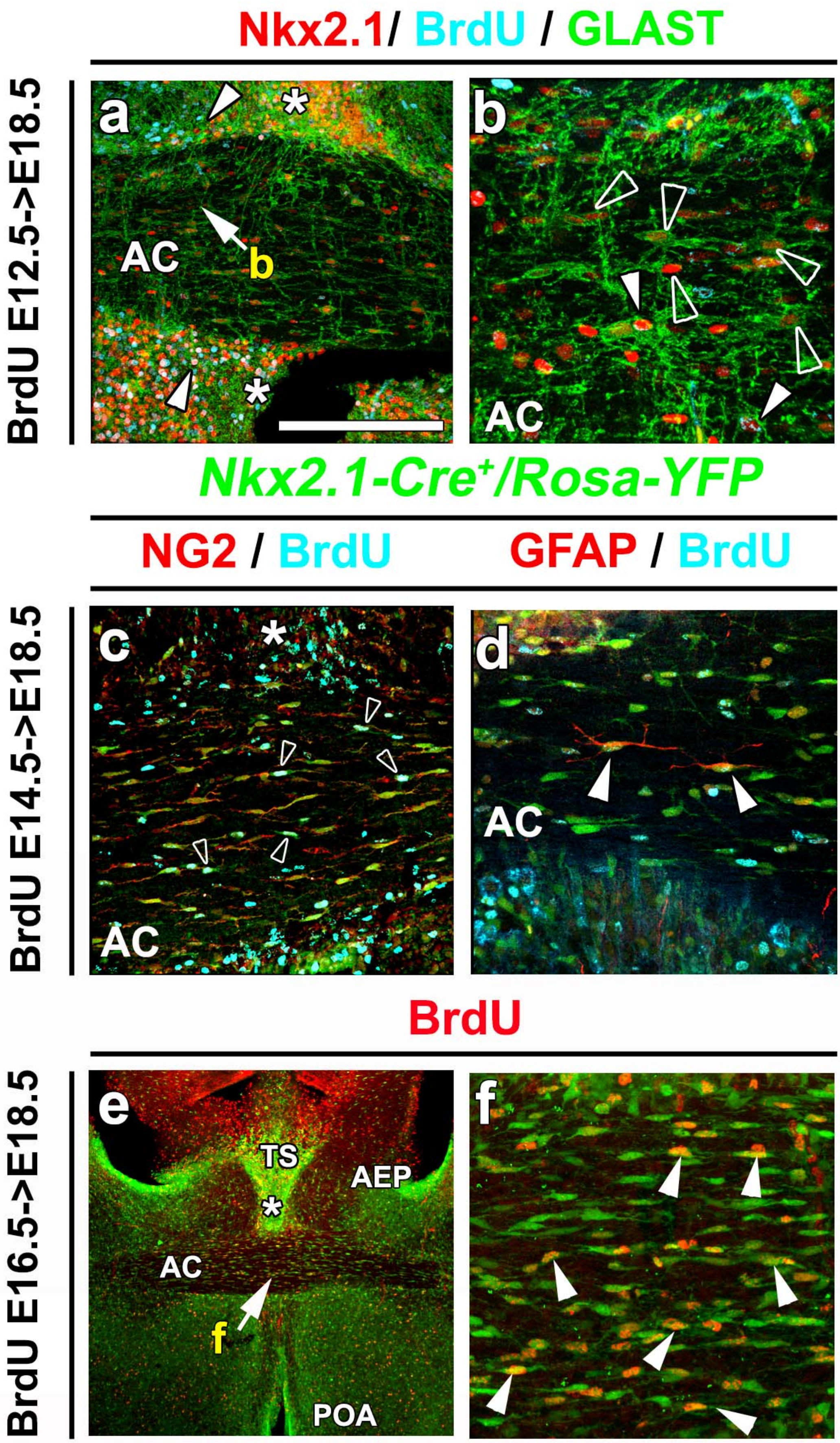

Supplementary Figure 3

Supplementary figure 3. Nkx2.1^{+} astroglia of the AC are generated between E14.5 and E16.5.

(a and **b)** Triple immunohistochemistry for Nkx2.1, 5-bromo2'-deoxy-uridine (BrdU), and GLAST on AC coronal sections from wild-type mice brains at E18.5 injected with BrdU at E12.5 (n=3). Only very few GLAST^{+} astroglia occupying the AC white matter at E18.5 appeared to be generated after E12.5 injections (solid arrowheads, **b**) while numerous GLAST^{+}/BrdU^{+} were visualized in the glia tunnel (*) (**a**).

(c-f) Double immunohistochemistry for BrdU and YFP (n=3) (**e-f**) and triple immunohistochemistry for NG2, BrdU and YFP (n=3) (**c**) and GFAP, BrdU and YFP (n=3) (**d**) on AC coronal sections from *Nkx2.1-Cre^{+}/Rosa-YFP* mice brains at E18.5 injected with BrdU at E14.5 (**c-d**) and E16.5 (**e-f**). The Nkx2.1-derived YFP^{+}/GFAP^{+} astroglia observed at E18.5 were generated from E14.5 to E16.5 as they were positively labeled with BrdU (solid arrowheads, **d** and **f**) in AC sections. **b** and **f** are a higher power views of the AC midline seen in **a** and **e** respectively.

(AEP) anterior entopeduncular area, **(POA)** preotic area, eminence, **(TS)** triangle septal nucleus. Bar = 675 µm in **e**; 100 µm in **a** and **c;** 60 µm in **d, f** and 50 µm in **b.**

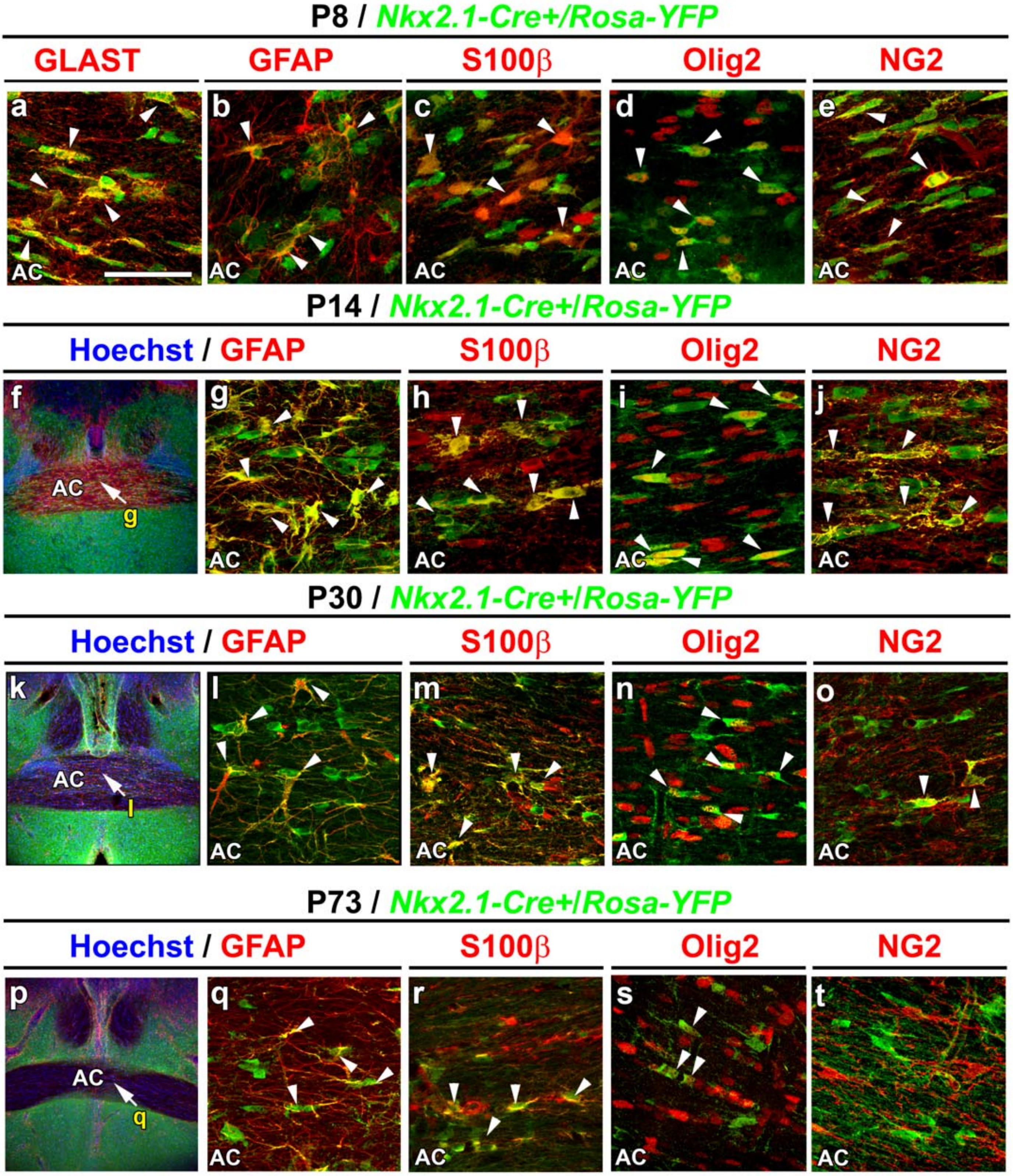

Supplementary Figure 4

Supplementary figure 4. Continued presence of *Nkx2.1-derived* astroglia in adult AC.

(**a-t**) Double immunohistochemistry for YFP and GLAST (n=3) **(a)**, YFP and GFAP (n=3) **(b, f-g**, **k-l** and **p-q)**, YFP and S100β (n=3) **(c, h, m** and **r)**, YFP and Olig2 (n=3) **(d, i, n** and **s)** and YFP and NG2 (n=3) **(e, j, o** and **t)** on anterior commissure **(AC)** coronal sections from *Nkx2.1-Cre^{+}/Rosa-YFP* mice at P8 **(a-e)**, P14 **(f-j)**, P30 **(k-o)** and in adults at P73 **(p-t)**. **g**, **l** and **q** are high power views of the AC pointed by an arrow in **f**, **k** and **p** respectively. (**a-j**) In the anterior commissure (AC) of *Nkx2.1-Cre^{+}/Rosa-YFP* mice brains at P8 (**a-e**) and P14 (**f-j**), numerous *Nkx2.1*-derived glial cells, co-expressing all types of glial markers (GFAP, S100β, Olig2 and NG2), were still present (depicted by yellow colocalization and solid arrowheads. **(k-o)** At P30, *Nkx2.1*-derived astroglial cells co-expressing GFAP and S100β were still present while only few *Nkx2.1*-derived polydendrocytes co-expressing NG2 remained in the AC. **(p-t)** In adults at P73, majority of the cells are positively co-stained for astrocytic marker GFAP and S100β, while only few were positively stained for the oligodendroglial marker Olig2. None of the cells were positively stained for the polydendrocyte marker NG2.

Cell nuclei were counterstained in blue with Hoechst **(f**, **k** and **p)**. Bar = 675 µm in **f**, **k** and **p**; 40 µm in **a-e**, **g-j**, **l-o** and **q-t**.

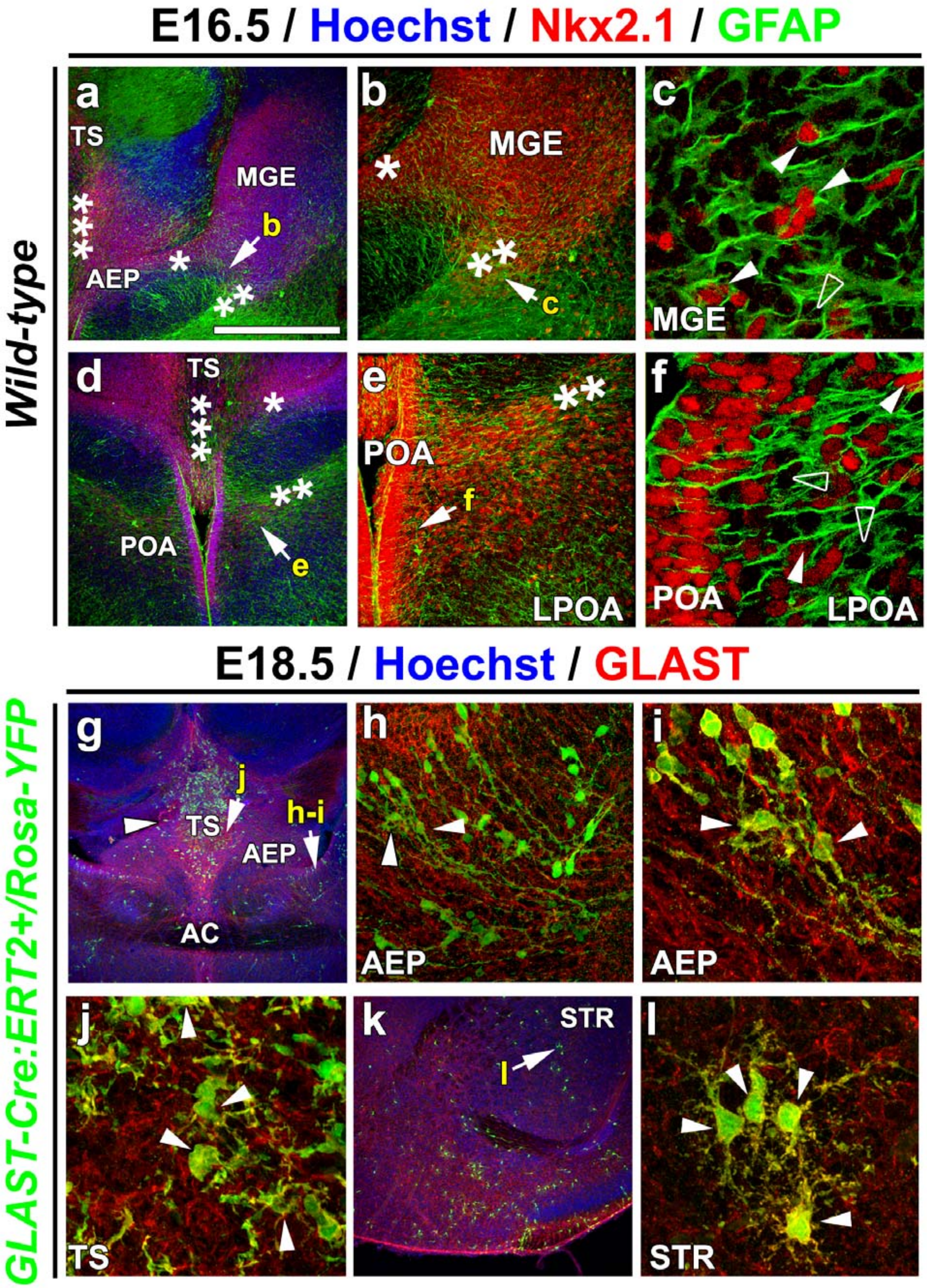

Supplementary Figure 5

Supplementary figure 5. Three subpallial sites of origin for *Nkx2.1*-derived astrocytes.

(a-f) Double immunohistochemistry for Nkx2.1 and GFAP (n=3) in coronal sections from wild-type mice at E16.5. **b**, **c, e** and **f** are high power views of the regions indicated by an arrow in **a**, **b**, **d** and **e** respectively. In E16.5 wild-type mice brains, several $Nkx2.1^+$ radial glial precursors (in red) expressing the GFAP (in green) were found in three different subpallial germinal regions, namely the triangular sepal nucleus **(TS)** **(a** and **d)**, the medial ganglionic eminence **(MGE)** **(b** and **c)** and the anterior entopeduncular/ preoptic area **(AEP/POA)** **(e** and **f)**. A flux of migrating $Nkx2.1^+$ postmitotic astroglial cells was observed between the MGE and the AEP (* in **a-b, d**), between the POA and the MGE (** in **a-b, d-e**), and between the TS and the AEP/POA (*** in **a** and **d**). **(c** and **f)** Solid arrowheads outline $Nkx2.1^+/GFAP^+$ postmitotic astroglia while open arrowheads indicate $Nkx2.1^-/GFAP^+$ astroglia.

(g-l) Double immunochemistry for YFP and GLAST (n=5) in coronal sections from *GLAST-Cre:ERT2*$^+$*/Rosa-YFP* mice at E18.5. **h-j** and **l** are high power views of the regions indicated by an arrow in **g** and **k**, respectively. **(g-l)** In E18.5 *GLAST-Cre:ERT2*$^+$*/Rosa-YFP* mice in which the Cre-mediated recombination was induced at E15 by a tamoxifen injection, several $GLAST^+$ post-mitotic astrocytes were observed to originate from the TS **(g** and solid arrowheads in **j)** and the AEP **(g** and solid arrowheads in **h** and **i)**. Astroglia were also observed to migrate to parenchymal regions such as the striatum **(STR)** **(k**, and solid arrowheads in **l)**.

Cell nuclei were counterstained in blue with Hoechst **(a**, **d**, **g** and **k)**. Colocalization between the green and the red channel is highlighted in yellow **(h-j** and **l)**. **(AC)** anterior commissure. Bar = 675 µm in **g** and **k**; 320 µm in **a** and **d**; 160 µm in **b, e** and **h** and 45 µm in **c**, **f**, **i**, **j** and **l**.

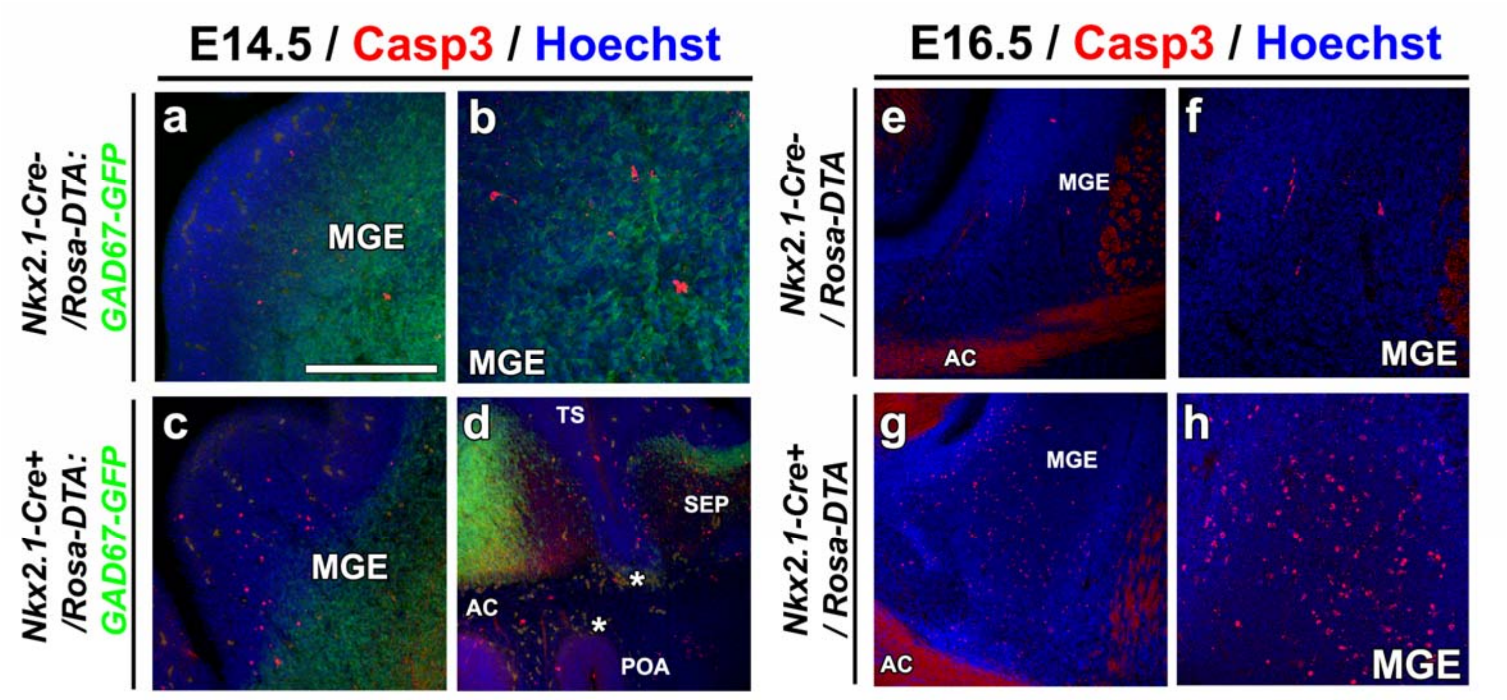

Supplementary Figure 6

Supplementary figure 6. Increased cell death in *Nkx2.1-Cre*$^{+}$*/Rosa-DTA* MGE at E16.5. **(a-h)** Immunochemistry for cleaved caspase 3 (Casp3) from control *Nkx2.1-Cre*$^{-}$*/Rosa-DTA:GAD67-GFP* (n=3) (**a-b**) and *Nkx2.1-Cre*$^{+}$*/Rosa-DTA:GAD67-GFP* (n=3) **(c-d)** mice at E14.5. **(e-h)** Immunochemistry for Casp3 on E16.5 coronal slices from *Nkx2.1-Cre*$^{-}$*/Rosa-DTA* (n=3) **(e-f)** and *Nkx2.1-Cre*$^{+}$*/Rosa-DTA* (n=3) **(g-h)** mice. Labeling with Casp3 revealed cell death of Nkx2.1^{+} progenitors in the medial ganglionic eminence **(MGE)** precursor regions in both control **(a-b)** and mutants **(c)** at E14.5. Also, we could observe increased Casp3 labeling in the anterior commissure **(AC)** region in the mutants **(d)**. The increased cell death was spread out in the MGE between E14.5 and E16.5 **(e-h)** since the diphtheria toxin took time to accumulate. **(POA)** preoptic area, **(SEP)** septum, **(TS)** triangular septal nucleus. Bar = 675 µm in **e** and **g**; 320 µm in **a, c, d, f** and **h**; 160 µm in **b**.

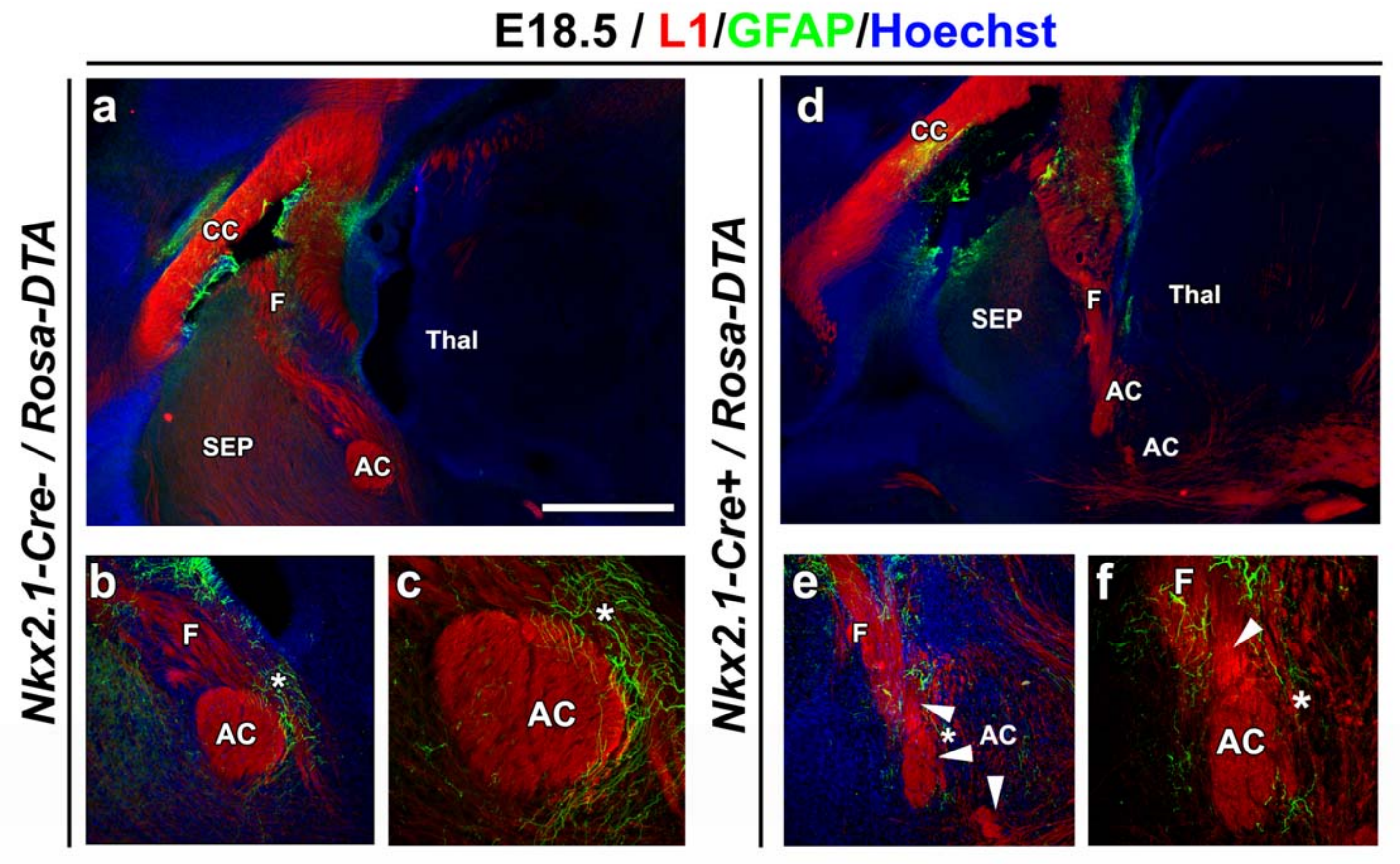

Supplementary Figure 7

Supplementary figure 7. AC defects in sagittal sections of E18.5 *Nkx2.1-Cre*$^{+}$*/Rosa-DTA* mice.

(a-f) Double immunochemistry for L1 and GFAP in sagittal sections from control *Nkx2.1-Cre*$^{-}$*/Rosa-DTA* (n=5) **(a-c)** and *Nkx2.1-Cre*$^{+}$*/Rosa-DTA* (n=5) **(d-f)** mice at E18.5. **b-c, e-f** are high-magnified views of the anterior commissure **(AC)** midline seen in **a** and **d**, respectively. In the mutant *Nkx2.1-Cre*$^{+}$*/Rosa-DTA* mice **(d-f)**, the AC axons were not as tightly bundled than in the control mice **(a-c)**. We observed evident defasciculation of the AC axons separated into two tracts (solid arrowheads in e), one projecting rostro-dorsally towards the septum. The axons in the fornix **(F)** were also disorganized. Moreover, the number of glia forming the tunnel structure **(* in b-c)** was clearly reduced in the mutant mouse **(* in e-f)**, and those that remained were misplaced.

Cell nuclei were counterstained in blue with Hoechst **(a-b** and **d-e)**. **(CC)** corpus callosum, **(SEP)** septum; **(Thal)** thalamus. Bar = 1350 µm µm in **a** and **d**, 320 µm in **b** and **e** and 160 µm in **c** and **f**.

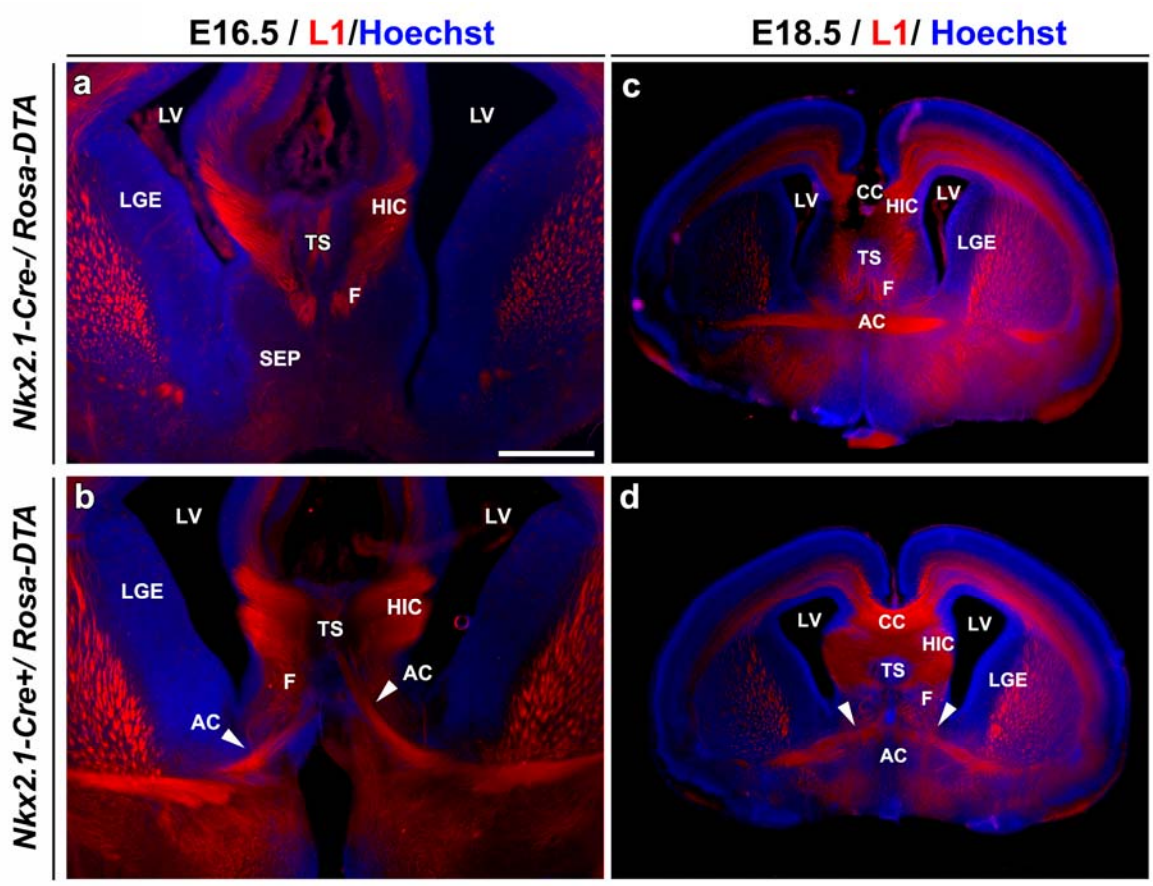

Supplementary Figure 8

Supplementary figure 8. Low-magnified views of AC in E16.5 and E18.5 *Nkx2.1-Cre$^+$/Rosa-DTA* mice.

(a-d) Immunochemistry for L1 in coronal sections from control *Nkx2.1-Cre$^-$/Rosa-DTA* **(a** and **c)** and *Nkx2.1-Cre$^+$/Rosa-DTA* **(b** and **d)** mice at E16.5 (n=3) **(a** and **b)** and E18.5 (n=4) **(c** and **d)**. **a-d** are low-magnified views of the telencephalon. (**c**) In *Nkx2.1-Cre$^-$/Rosa-DTA* control mice, L1$^+$ commissural axons crossed the anterior commissure **(AC)** midline and grew towards the contralateral cortex. (**b** and **d**) By contrast, in the mutant *Nkx2.1-Cre$^+$/Rosa-DTA* mice, the majority of AC commissural axons did not cross the midline and form two large ectopic bundles of axons on either ipsilateral side of it (solid arrowheads). In the mutant mice (b and d) contrary to the control mice (a and c), the axons of the dorsal tract were misrouted in the triangular septal nucleus **(TS)** or in the fornix **(F)**. **(HIC)** hippocampal commissure, **(LGE)** lateral ganglionic eminence, **(LV)** lateral ventricle, **(SEP)** Septum. Bar = 2700 µm in **a** and **b**, 1350 µm in **c** and **d**.

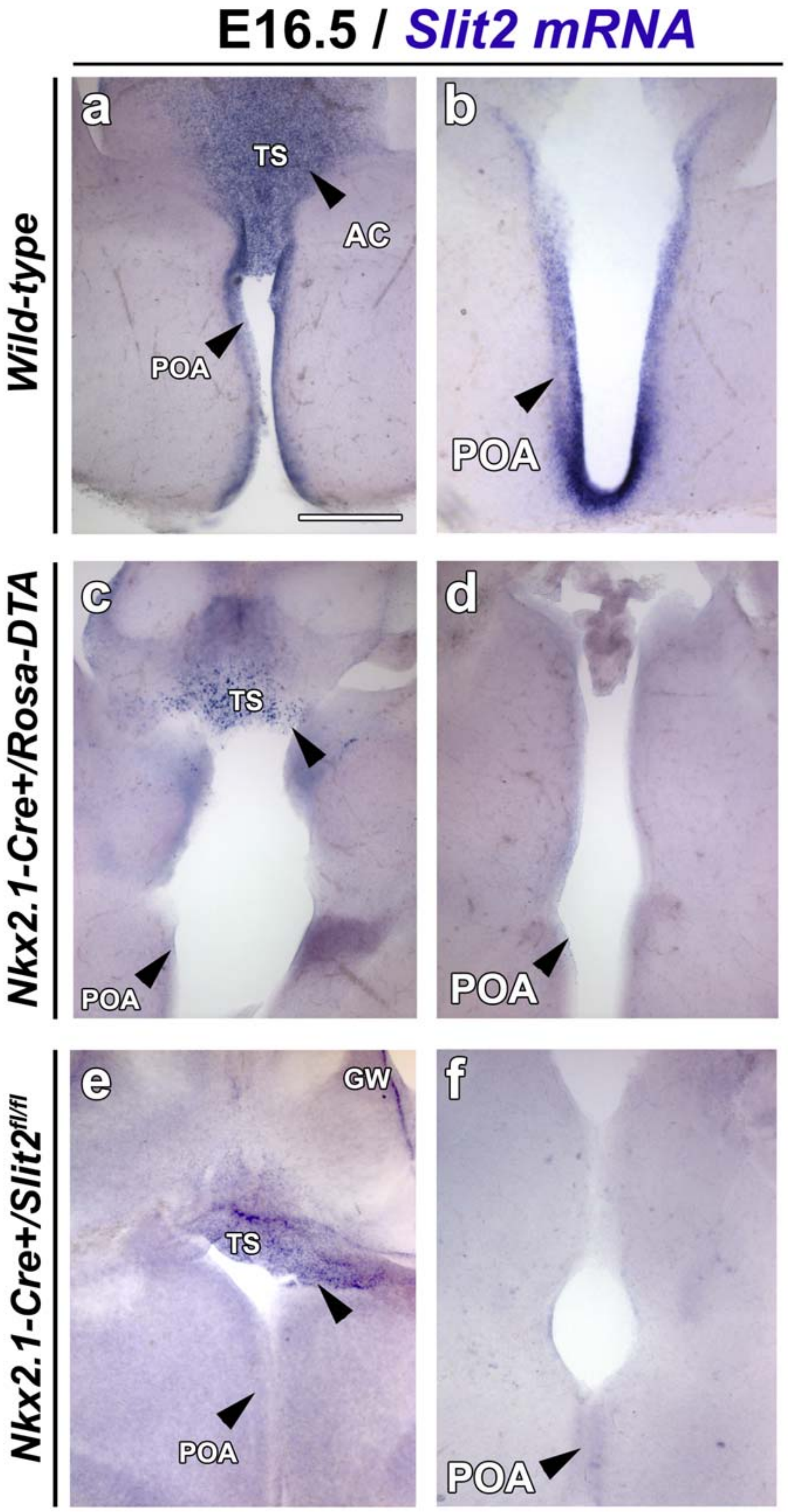

Supplementary Figure 9

Supplementary figure 9. Strong decrease in *Slit2* in Nkx2.1-Cre^{+}/Rosa-DTA and Nkx2.1-Cre^{+}/Slit2$^{fl/fl}$ mice.

(a-f) *In situ* hybridization for *Slit2* on coronal slices from wild-type (n=5) **(a-b)**, *Nkx2.1-Cre^{+}/Rosa-DTA* (n=5) **(c-d)**, and *Nkx2.1-Cre^{+}/Slit2$^{fl/fl}$* (n=3) **(e-f)** mice at E16.5. **(a-b)** In wild-type mice, the Nkx2.1^{+} precursors of the preoptic area **(POA)** and the triangular septal nucleus **(TS)** strongly expressed the mRNA for the repellent guidance molecule Slit2. (**c-f**) In *Nkx2.1-Cre^{+}/Rosa-DTA* **(c-d)** and *Nkx2.1-Cre^{+}/Slit2$^{fl/fl}$* (**e-f**) mice, the expression of *Slit2* mRNA is reduced in the TS and the POA compared to control mice (**a-b**). **(AC)** anterior commissure. Bar = 500 µm in **a-f.**
